# Phase Behavior of Alkyl Ethoxylate Surfactants in a Dissipative Particle Dynamics Model

**DOI:** 10.1021/acs.jpcb.2c08834

**Published:** 2023-02-14

**Authors:** Richard L. Anderson, David S. D. Gunn, Tseden Taddese, Ennio Lavagnini, Patrick B. Warren, David J. Bray

**Affiliations:** The Hartree Centre, STFC Daresbury Laboratory, Warrington WA4 4AD, United Kingdom

## Abstract

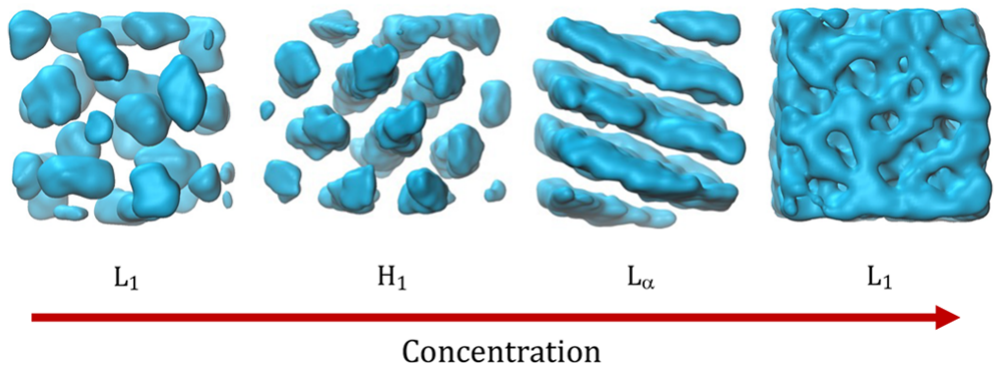

We present a dissipative particle dynamics (DPD) model capable of capturing the liquid state phase behavior of nonionic surfactants from the alkyl ethoxylate (C_*n*_E_*m*_) family. The model is based upon our recent work [Anderson et al. *J. Chem. Phys.***2017**, *147*, 094503] but adopts tighter control of the molecular structure by setting the bond angles with guidance from molecular dynamics simulations. Changes to the geometry of the surfactants were shown to have little effect on the predicted micelle properties of sampled surfactants, or the water–octanol partition coefficients of small molecules, when compared to the original work. With these modifications the model is capable of reproducing the binary water–surfactant phase behavior of nine surfactants (C_8_E_4_, C_8_E_5_, C_8_E_6_, C_10_E_4_, C_10_E_6_, C_10_E_8_, C_12_E_6_, C_12_E_8_, and C_12_E_12_) with a good degree of accuracy.

## 1 Introduction

In a recent publication,^1^ we described a coarse-grained dissipative particle dynamics (DPD) “force field” for chemistries in common use in the fast-moving consumer goods sector. Here we used a molecular fragmentation strategy in which DPD “beads” represent chemical subgroups and moieties typically comprising 1–3 “heavy” atoms such as carbon, oxygen, etc. With bond lengths heuristically based on the number of heavy atoms in the bonded pair and equilibrium bond angles left unbiased (i.e., 180°), the main focus of the study was on parametrizing the nonbonded bead–bead pair interactions against experimental data comprising water–octanol partition coefficients (log *P* values) and liquid phase densities (at 25 °C). Despite the simplifying assumptions, the resulting model successfully reproduced the critical micelle concentrations (CMCs) of several commercially relevant nonionic, alkyl ethoxylate (C_*n*_E_*m*_) surfactants and recovered the correct trend for micelle mean aggregation number (*N*_agg_).

In this work we probe the ability of this force field to reproduce the liquid state *phase behavior* of these surfactants. We additionally include a more rigorous consideration of the bond angles, motivated by our recent work on waxing in alkanes and aromatic hydrocarbons,^2,3^ and refine the parameters for two key bead–bead interactions. The revised model captures the phase behavior of the selected surfactants to a reasonable extent, while maintaining performance for CMCs and log *P* values as in the original work.

Micelle formation and the phase behavior of alkyl ethoxylate surfactants have received significant attention from the simulation community over the past two decades, with progress being made using both atomistic and coarse-grained molecular dynamics^4−11^ and DPD simulation approaches.^1,12−15^ This in part is not only due to the importance of these surfactants in many applications (e.g., cosmetics, textiles, agriculture, paper, and textile industries^16^) and the wide range of self-assembled structures formed by these molecules but also due to the abundance of experimental data available with which to validate simulated results, including, but by no means limited to, Clunie et al.,^17^ Mitchell et al.,^18^ Sakya et al.,^19^ Garavito and Ferguson-Miller,^20^ and Swope et al.^21^

We organize the paper by first outlining the phase behavior of alkyl ethoxylate surfactants that we are targeting. Then we define the DPD model employed for this work. Following this, we explore the CMCs and *N*_agg_ of three alkyl ethoxylates in addition to verifying the new model via log *P*. We then examine the performance of the model reproducing the phase behavior of nine C_*n*_E_*m*_ surfactants in the C_8_, C_10_, and C_12_ families at 25 °C. Appendix A provides technical details of the approach to parametrize the bond angle potentials and Appendix B explores micelle aggregation behaviors observed. In the Supporting Information we also provide a quantification of the phase behavior resulting from the DPD models of Johnston et al.^22^ and Lavagnini et al.^23^

## 2 Phase Behavior of Alkyl Ethoxylates

Alkyl ethoxylate surfactants can form a wide array of self-assembled structures when mixed with water. At low surfactant concentrations C_*n*_E_*m*_ molecules aggregate to form small micelles provided the CMC is exceeded. As the concentration of surfactant is increased, the micelles become more elongated, although for some, particularly where both the alkane and ethoxylate regions are quite long, micelles remain spherical over a large range of concentration. As the concentration increases further, the micelle behavior can vary significantly depending on the chain lengths of the C and E blocks, the ratio of *n*:*m*, and the temperature of the solution. Eventually, packing considerations force the micelles to order and/or restructure, and a variety of lyotropic liquid crystal phases appear. Finally, as the water content decreases, the system usually reverts to an isotropic liquid phase again.

**Figure 1:**
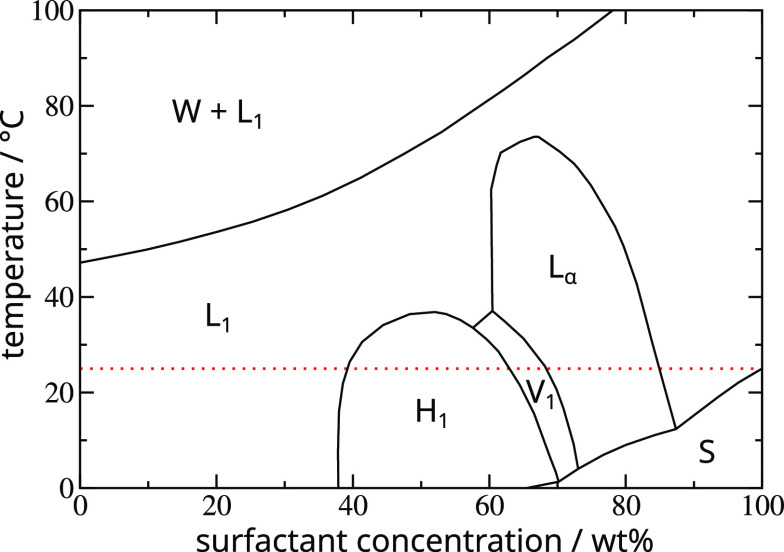
Phase diagram for C_12_E_6_ + water in the temperature–composition plane. The red dotted line is the 25 °C isotherm. Reproduced with permission from ref (18). Copyright 1983 Royal Society of Chemistry.

The phase diagram for C_12_E_6_, shown in Figure 1, is typical. We focus on the 25 °C isotherm (red dotted line) which is pertinent to this study. At concentrations <40 wt % surfactant, this system displays an isotropic liquid (L_1_) phase in which the micelles exhibit no long-range order. Between 40 and 62 wt %, a hexagonal liquid crystal phase (H_1_) appears, where rod-like surfactant aggregates pack with long-range hexagonal order. There is next a small concentration window between 62 and 68 wt % in which a bicontinuous gyroidal cubic phase (V_1_) is present. From 68 to 85 wt % a lamellar phase (L_α_) is found, which is formed from stacked sheet-like aggregates. Above 85 wt %, C_12_E_6_ exhibits a re-entrant L_1_ isotropic liquid phase, in this case formed by entangled and bridged micelles, before solid surfactant starts to precipitate out (region indicated by S on the diagram). We note that the transitions between the various liquid crystal phases are weakly first-order, so strictly speaking narrow two-phase coexistence regions should be drawn between phases in Figure 1, with the tops of the phase “domes” being azeotropes. This fine detail is not resolved in our simulations and we do not pursue it further. Also, we note that at low to moderate concentrations but well above the 25 °C isotherm, a liquid–liquid demixing transition or “clouding” region (W+L_1_) is also seen in the C_12_E_6_ system. This is a typical feature for alkyl ethoxylate surfactants and reflects the demixing tendency in water of the ethoxylate chains with increasing temperature, seen for instance in the existence of a lower critical solution temperature for the water-soluble poly(ethylene oxide) (PEO) polymer.^24^ Finally, depending upon the surfactant, additional liquid crystal phases can be observed. An example relevant for this work is the cubic micellar phase (I_1_) present for C_12_E_12_ between the L_1_ and H_1_ phases (see Figure 5).

## 3 DPD Model for C_*n*_E_*m*_ Surfactants

### 3.1 Atom to Bead Mapping

In our approach, DPD beads represent molecular fragments comprising *n* = 1–3 “heavy atoms” (e.g., carbon and oxygen) with the exception of water (H_2_O), which is treated supermolecularly. For this, we set the “mapping number” *N*_*m*_ = 2, where *N*_*m*_ is the number of water molecules per water bead.^25^ Following well-established protocols,^26^ we use the cutoff distance (*r*_*c*_) for the soft repulsive interaction between water beads as the unit of distance and set the dimensionless water bead number density representing pure water to ρ*r*_*c*_^3^ = 3 and the corresponding repulsion amplitude to *A*_*ij*_ = 25. We note that this choice does not match the compressibility of water in our model, but we are not alone in regarding an exact match as unnecessarily restrictive.^27^ Further information can be found in Anderson et al.^28^ Then, with Avogadro’s constant *N*_A_ and the molar volume of water *V*_*w*_ ≈ 18 l mol^–1^, the volume occupied by one mole of DPD “volume elements” is *N*_A_*r*_*c*_^3^ = ρ*r*_*c*_^3^*N*_*m*_*V*_*w*_ ≈ 0.108 × 10^–3^ m^3^. From this, we infer *r*_*c*_ ≈ 5.64 Å.

Because C_*n*_E_*m*_ surfactants comprise an alkyl (block) chain of length *n* and an ethoxylate (block) chain of length *m*, usually terminated by an alcohol group (−OH), they are readily accommodated in the model by representing the alkane functionality as a chain of CH_2_CH_2_ (alkyl) beads with a terminal CH_3_ (methyl) bead and the ethoxylate counterpart by a chain of CH_2_OCH_2_ (ether) beads with a terminal CH_2_OH (alcohol) bead. The resulting coarse-grained C_*n*_E_*m*_ surfactant model is therefore
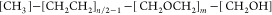
where the square brackets denote the different bead types. There are therefore five bead types in our model, corresponding to 2H_2_O, CH_3_, CH_2_CH_2_, CH_2_OCH_2_, and CH_2_OH. Because the bead counts with this strategy have to be integers, *n* must be divisible by two, and this limits us to representing alkyl ethoxylates with an even number of carbon atoms in the alkyl chain. In our work on alkane waxes (although not implemented here),^2^ this restriction is lifted by introducing small DPD beads to represent single CH_2_ groups. Another approach would be to allow for the possibility of a terminating CH_3_CH_2_ bead. Either would be a valid extension of the work presented here. We now turn to the interactions (nonbonded and bonded) between these bead types.

### 3.2 Nonbonded Interactions

The nonbonded interactions are defined by standard DPD pairwise soft repulsions^26,29^ of the form *U*_*ij*_ = *A*_*ij*_*R*_*ij*_(1 – *r*_*ij*_/*R*_*ij*_)^2^ for *r*_*ij*_ ≤ *R*_*ij*_ and *U*_*ij*_ = 0 for *r*_*ij*_ > *R*_*ij*_, where *A*_*ij*_ is the repulsion amplitude of the DPD conservative force, *R*_*ij*_ is the cutoff distance, and *r*_*ij*_ = |*r⃗*_*j*_ – *r⃗*_*i*_| is the separation between beads *i* and *j* located at *r⃗*_*i*_ and *r⃗*_*j*_, respectively. We set *A*_*ij*_ = 25 and *R*_*ij*_ = *r*_*c*_ for water beads.

This choice implies *k*_B_*T* = 1 is the unit of thermal energy in DPD, where *k*_B_ is the Boltzmann constant and *T* the temperature. However, in an NVT or NPT simulation this is only required to specify the thermostat, and taking it too literally would be a mistake. Keeping *A*_*ij*_ constant while varying *T* would fail to represent, for instance, the declining solubility of the ether groups in water with increasing temperature, which is the root cause of the clouding phenomenon mentioned earlier. Our model is therefore strictly limited to the choice made for the parametrization temperature (25 °C). Parametrization at other temperatures can proceed likewise, if data are available, and the results are used to interpolate the parameters to intermediate temperatures. However, this goes beyond the scope of the current paper, and it should be addressed in a future work.

As in our previous work,^1^ we use the self-interaction cutoffs *R*_*ii*_ to capture the contribution of the molecular fragments to the overall molar volumes (here and below *i* denotes bead type, not individual beads). For this, the Durchschlag and Zipper rules were used to assign *R*_*ii*_^3^ values for different beads in proportion to their fragment molar volumes,^30^ taking the molar volume of water as a reference for which *R*_*ii*_^3^ ≡ *r*_*c*_^3^ = 1. Then, between dissimilar bead types we use a simple arithmetic “mixing rule” *R*_*ij*_ = (*R*_*ii*_ + *R*_*jj*_)/2.

For the repulsion amplitudes we use, for the most part, the values of *A*_*ij*_ determined in our earlier work, obtained by fitting to experimental water–octanol partition coefficients (log *P* values), mutual solubilities, and liquid density data, for a range of small molecules.^1^

Where this study differs from the parameters of our earlier work is in the interactions between the ether bead CH_2_OCH_2_ and the alkane beads; we reduce these from *A*_*ij*_ = 28.5 to 24.2. The motivation for this change is based on the experimental observations that C_12_E_6_ should transition from an L_α_ to an L_1_ phase between 84 and 88 wt %, and C_8_E_4_ should show only an L_1_ phase across the entire concentration range. With the original choice, the L_α_ phase of C_12_E_6_ persists to too high concentrations and an H_1_ phase appears for C_8_E_4_. Note that this change in *A*_*ij*_ from 28.5 to 24.2 brings this parameter in much closer agreement with the value presented by Lavagnini and co-workers, who trained a DPD model for alkyl ethoxylates using single points interaction based on *ab initio* molecular electrostatic potential surfaces.^23^ It is worth noting that the transition from L_α_ to L_1_ in C_12_E_6_ is highly sensitive to the aforementioned *A*_*ij*_ parameter. For example, increasing the repulsion amplitude between the ether and the alkane beads (from *A*_*ij*_ = 24.2) to *A*_*ij*_ = 25.0 results in an L_α_ to L_1_ transition at about 90 wt % (versus ∼85 wt %). Table 1 lists the values of *R*_*ij*_ and *A*_*ij*_ for all beads used in this study.

**Table 1:** Repulsion Amplitudes and Cutoff Distances Used in the Present Study

bead *i*	bead *j*	*A*_*ij*_	*R*_*ij*_
2H_2_O	2H_2_O	25.0	1.0000
2H_2_O	CH_2_CH_2_	45.0	1.0370
2H_2_O	CH_3_	45.0	0.9785
2H_2_O	CH_2_OCH_2_	24.0	1.0580
2H_2_O	CH_2_OH	14.5	0.9900
CH_2_CH_2_	CH_2_CH_2_	22.0	1.0740
CH_2_CH_2_	CH_3_	23.0	1.0155
CH_2_CH_2_	CH_2_OCH_2_	24.2^a^	1.0950
CH_2_CH_2_	CH_2_OH	26.0	1.0270
CH_3_	CH_3_	24.0	0.9570
CH_3_	CH_2_OCH_2_	24.2^a^	1.0365
CH_3_	CH_2_OH	26.0	0.9685
CH_2_OCH_2_	CH_2_OCH_2_	25.5	1.1160
CH_2_OCH_2_	CH_2_OH	25.0	1.0480
CH_2_OH	CH_2_OH	14.0	0.9800

a Previously 28.5 in Anderson et al.^1^

To complete the description, the cutoff distance for the dissipative and random forces required in the DPD method was assigned equal to the maximum cutoff distance in the system (i.e., set as the maximum value of *R*_*ii*_), and the dissipative friction amplitude was set at γ = 4.5.

### 3.3 Bonded Interactions

A simple harmonic potential *U*_*ij*_^*B*^ = *k*_*b*_(*r*_*ij*_ – *r*_0_)^2^ is used for the bonds between connected DPD beads. As in our earlier work,^1^ nominal bond lengths are set according to the number of heavy atoms *n*_*i*_ and *n*_*j*_ in the bonded beads,^1^ as *r*_0_ = 0.1(*n*_*i*_ + *n*_*j*_) – 0.01, and a single bond constant *k*_*b*_ = 150 (DPD units) was adopted throughout. For CH_2_CH_2_ bonded pairs this results in *r*_0_ = 0.39, and the equivalent for CH_2_OCH_2_ beads is *r*_0_ = 0.59. In real units this corresponds to 2.2 and 3.3 Å, respectively. Note that in our approach the standard DPD (nonbonded) repulsions are retained between all beads, including directly bonded pairs. This has the effect of increasing the bond length (in addition to fluctuation and correlation effects) above the nominal value indicated by *r*_0_. Thus, between pairs of bonded CH_2_CH_2_ beads this results in a mean bond length of 0.45 DPD units, or 2.5 Å, while between pairs of bonded CH_2_OCH_2_ beads we obtain a mean bond length of 0.66 DPD units, or 3.8 Å. Corrections to the *r*_0_ values can be determined using eq 1 of the Supporting Information from Bray et al.^2^ or from direct measurement from simulation. For the CH_2_CH_2_ pairs, this value is in good alignment with known bond lengths, while for CH_2_OCH_2_ pairs the result is higher than that reported for the analogous ether beads from the Martini force field.^31^ In the present work we do not refine the *r*_0_ values as to do so would significantly impact the underlying parametrization based upon fitting to liquid densities and log *P* values.^1^

We introduce bond rigidity by including a harmonic angular potential between pairs of bonds. We adopt the three-body angular potential used by Smit and collaborators,^32,33^ viz. *U*_*ijk*_^*A*^ = ^1^/_2_*k*_*a*_(θ_*ijk*_ – θ_0_)^2^ where θ_*ijk*_ is the angle between adjoining bonds. Unlike our previous approach where we used θ_0_ = 180° and *k*_*a*_ = 5 (DPD units) for all angles, we here set θ_0_ and *k*_*a*_ with the aid of atomistic simulations of pure systems of alkane, PEO, and C_*n*_E_*m*_ molecules. The details are presented in Appendix A, and the angle parameters thus generated are reported in Table 2.

**Table 2:** Bond Angle Parameters Used in the Present Study

bead *i*	bead *j*	bead *k*	*k*_*a*_	θ_0_ (deg)
CH_3_	CH_2_CH_2_	CH_2_CH_2_	30	180
CH_2_CH_2_	CH_2_CH_2_	CH_2_CH_2_	30	180
CH_2_CH_2_	CH_2_CH_2_	CH_2_OCH_2_	15	180
CH_2_CH_2_	CH_2_OCH_2_	CH_2_OCH_2_	15	145
CH_2_OCH_2_	CH_2_OCH_2_	CH_2_OCH_2_	15	132
CH_2_OCH_2_	CH_2_OCH_2_	CH_2_OH	15	145

## 4 Simulation Protocols

All DPD simulations were performed using the DL_MESO simulation package.^34^ Reduced units are used throughout in which all DPD beads have unit mass, the temperature *k*_B_*T* = 1, and the base length *r*_*c*_ = 1 (i.e., cutoff for water bead self-interaction) as indicated in the previous section. A DPD time step of 0.01 (in reduced units) was adopted throughout, and trajectory data were collected every 10 DPD time units (equivalent to 10^3^ time steps). Simulations were performed under constant pressure conditions (NPT ensemble) using the Langevin piston implementation of Jakobsen.^35^ The pressure was set to *P* = 23.7 (in DPD units) which corresponds to the pressure in an NVT simulation box of pure water at a water bead density ρ*r*_*c*_^3^ = 3 with interaction parameters *A*_*ij*_ = 25 and *R*_*ij*_ = 1.0 (as is typical for DPD simulation).^26^ Trajectory data files were analyzed using the UMMAP analysis package to obtain results unless otherwise stated.^36^

### 4.1 Determining Phase Behavior

The phase behavior of the nonionic surfactants of the C_*n*_E_*m*_ family were explored using cubic simulation boxes containing 81000 beads (box size ≈30^3^ DPD units under the imposed constant *P* = 23.7 conditions). Each simulation was started with molecules arranged randomly and was run for a minimum of 2 × 10^5^ DPD time units (20 million steps). Surfactant mesophases were identified by a combination of visual inspection and the orientational order parameter previously introduced by Warren et al.^12^ For the latter, isosurfaces are constructed around the alkane chains of the surfactant molecules. The isosurface normals are then extracted and used to construct as an order parameter the second moment **M** = ⟨*n⃗n⃗*⟩_*p*_ of the isosurface normal distribution *p*(*n⃗*). Because **M** is a symmetric second-rank tensor, it can be characterized by three real eigenvalues (μ_*i*_), which we rank by magnitude and monitor as a function of time. We note that by construction ∑_*i*_μ_*i*_ = tr **M** = 1. The relative magnitudes of the eigenvalues can be used to identify the phase present in the simulation boxes. For isotropic phases such as L_1_, all three eigenvalues ≈1/3 since the isosurface normals point equally in all three dimensions. For a hexagonal (H_1_) phase, one eigenvalue is typically small and the other two are ≈1/2 because the isosurface normals are predominantly confined to a two-dimensional plane. Finally, for a lamellar (L_α_) phase, two eigenvalues are small, and one is ≈1 since the isosurface normals mainly project along a single axis. Warren et al.^12^ set quite stringent cutoff values for μ_*i*_ corresponding to different phases in their previous work. These worked well for the highly idealized surfactant model used in that study (i.e., surfactant molecules represented by dimers) because the mesophases formed were highly ordered and stable. We choose not to employ these same values and instead focus upon the trends in the magnitudes of the eigenvalues as a function of concentration. The resulting mesophases in this work are subject to undulations, bridging, and imperfections that make using prespecified, hard set, cutoffs difficult.

**Figure 2:**
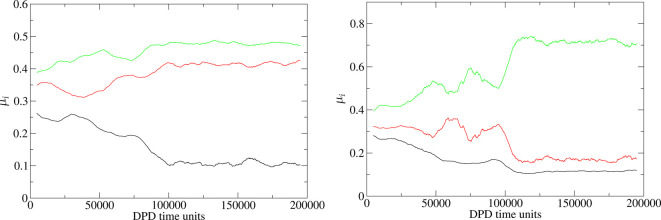
Eigenvalues μ_*i*_ of the orientational order parameter **M** for C_12_E_8_ as a function of time measured in DPD time units, displaying an H_1_ phase (left) at 55 wt % and an L_α_ phase (right) at 85 wt %. In both cases equilibration is reached after *O*(10^5^) DPD time units. Eigenvalues are presented as running averages over a window of 100 DPD time units. The corresponding raw, nonaveraged, data can be very noisy as the mesophases undulate and structures bridge and branch between sampled time frames resulting in significant fluctuations in the instantaneous eigenvalues.

The time taken for the various mesophases to form in the simulations varies significantly and depends somewhat on the concentration and distance from a phase boundary. As a broad rule of thumb, evidence for the growth of certain phases can be observed in the evolution of the eigenvalues μ_*i*_, with these settling down typically after 10^5^ DPD time units. However, H_1_ behavior often emerges over a longer time scale, and we observed a few cases in which more than 2.5 × 10^5^ DPD time units were required. Early work on DPD by Groot, Madden, and Tildesley also finds slow growth of these phases.^37^Figure 2 shows typical examples of the equilibration of the eigenvalues μ_*i*_ for systems exhibiting H_1_ and L_α_ phases.

For each surfactant the phase behavior was sampled over a concentration range of 20–90 wt % (with an exception for C_12_E_6_ for which we sample to 97 wt % in Figure 5) in intervals of 10 wt % for an initial pass. Where phase transitions were observed between two intervals, further simulations were performed halfway between the two points. In general, simulations were not performed below the experimental Krafft boundary where solid surfactant would precipitate out (regions labeled as “S” in the phase diagrams). However, this is only relevant for long chain surfactants such as C_12_E_12_. In our previous work,^2^ we have shown that it is possible to observe freezing (waxing) of long chain alkanes with a DPD model through careful consideration of the geometrical constraints (bonds and angles). We did check whether pure C_12_E_12_ freezes in the current model, but with the present parameter set it does not. The reason for this is most likely 2-fold. First, in the work of Bray et al.^2^ we not only set the angle constraints with help from atomistic simulation (as we do in this paper) but also employed a much stiffer spring constant (5000 *k*_B_*T* rather than 150 *k*_B_*T* in this work) which provides much tighter control of the bond lengths. We steer away adopting such stiff spring constants in the present model to avoid damaging the underlying parametrization to liquid phase densities and log *P*.^1^ However, we did test a C_12_E_12_ model where we made this change (i.e., increased the bond stiffness), and we were still unable to reproduce the freezing transition. This brings us to our second potential consideration which is the ethylene oxide chains themselves potentially require further optimization, e.g., by further refinement of the bonded interactions and/or by adopting a many-body approach to make the interactions locally dependent on the water content. We do not consider this any further in this study. More details of the C_12_E_12_ simulations are given in the Supporting Information.

**Figure 3:**
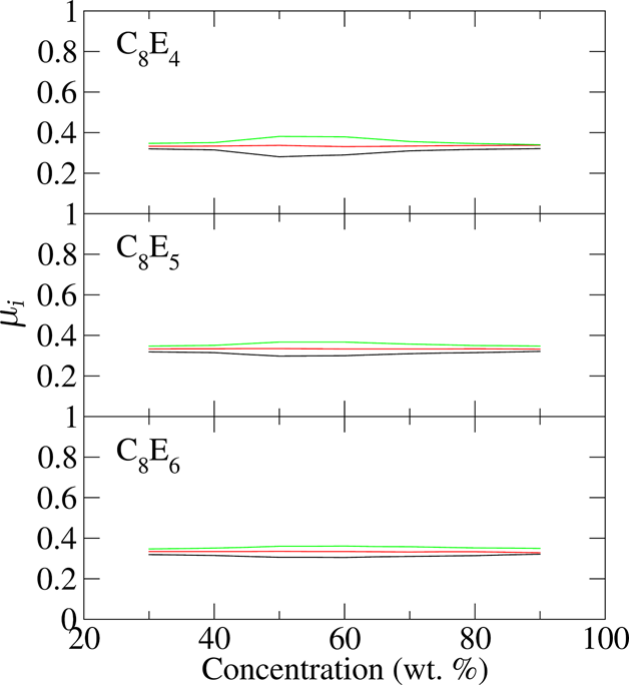
Eigenvalues of the second moment of the isosurface normal distribution, as a function of concentration, for C_8_E_4_, C_8_E_5_, and C_8_E_6_.

**Figure 4:**
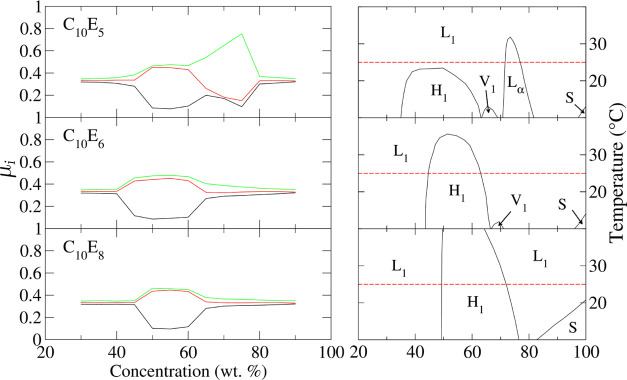
Eigenvalues (left) as a function of concentration for C_10_E_5_, C_10_E_6_, and C_10_E_8_. The corresponding experimental phase diagrams are shown right, with the 25 °C isotherms indicated (red dashed lines). Experimental phase diagrams are reproduced with with permission from ref (46). Copyright 1998 Elsevier.

**Figure 5:**
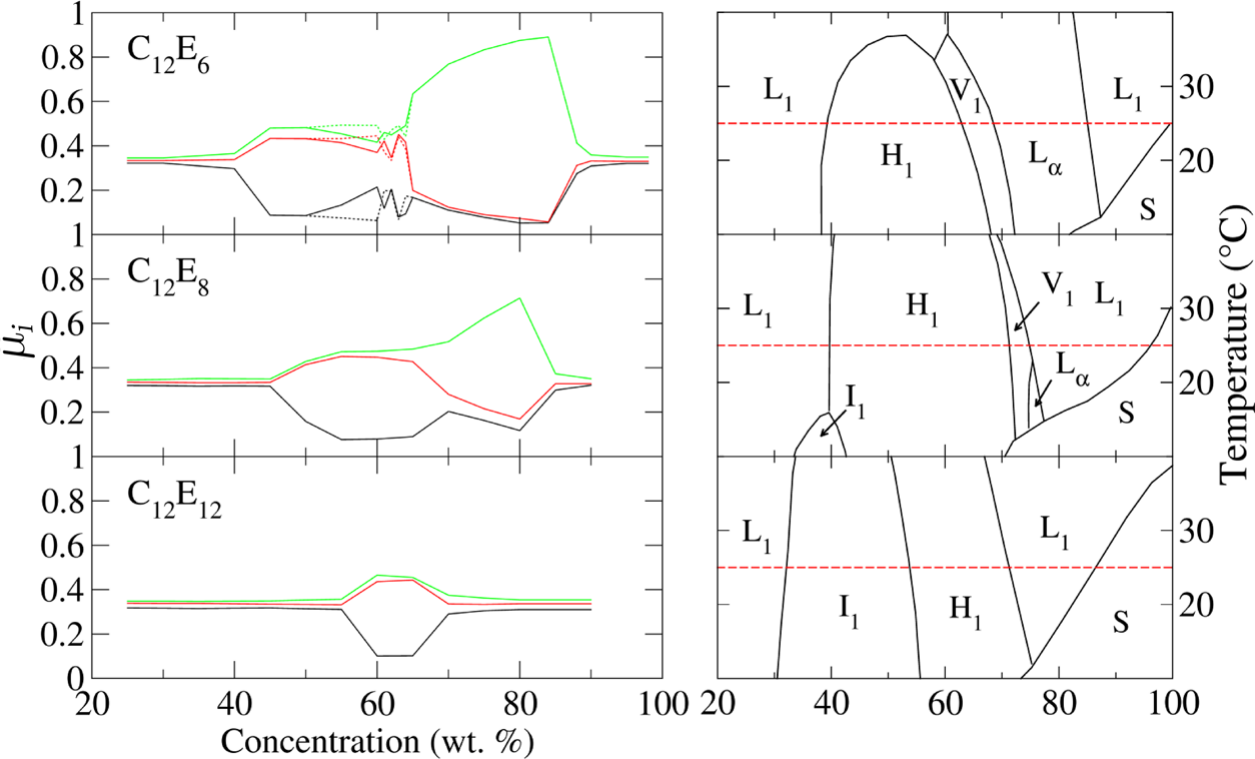
Eigenvalues (left) as a function of concentration for C_12_E_6_, C_12_E_8_, and C_12_E_12_. Dotted lines in the plots of the eigenvalues of C_12_E_6_ correspond to repeated simulations (see main text). The corresponding experimental phase diagrams are shown on the right, with the 25 °C isotherms indicated (red dashed lines). Experimental phase diagrams for C_12_E_6_ and C_12_E_8_ are reproduced with permission from ref (18). Copyright 1983 Royal Society of Chemistry.

Table 3 outlines the criteria adopted for assigning phases to simulation outputs. We now explain this in more detail. Determination of the H_1_ and L_α_ phases is straightforward as the isosurface normal eigenvalues are robust in these cases. Here, distinct separation of the eigenvalues clearly indicates the phase (see Figures 3–5). The L_1_, I_1_ and V_1_ phases are harder to assign by eigenvalue alone and require visual inspection of the microstructure. In our studies, V_1_ phases exist only between H_1_ and L_α_ phases. Hence they broadly appear as a transition where μ_1_ < μ_2_ < μ_3_. We are unsure how these eigenvalues would appear in a system that transitions from H_1_ to V_1_ and directly to L_1_. As such it is difficult to provide generic guidance. The L_1_ to I_1_ transition in the low concentration regime is also subtle, with it being the case that μ_1_ ≈ μ_2_ ≈ μ_3_ in both phases. This is because both phases have broadly spherical micelles and as such approximately equal eigenvalues. To differentiate between these phases one should therefore visually inspect the microstructure and look for positional order in the micelles. At high concentrations we encounter L_1_ phases where again μ_1_ ≈ μ_2_ ≈ μ_3_. However, at this point we no longer have small micelles. Rather we see a disordered isotropic microstructure composed of randomly branched and connected structures that fill the space of the simulation cell entirely. As the structure is random, the isosurface normals are equally orientated in all three spatial directions and again the corresponding eigenvalues are broadly equal. To aid future work, we present exemplar structures from each phase encountered in this study in the Supporting Information.

**Table 3:** Criteria for Phase Identification from Simulation

phase	no. of surfactant aggregates	shape of aggregate	isosurface normal eigenvalues
L_1_ (low concn)	many	small	μ_1_ ≈ μ_2_ ≈ μ_3_ ≈ 1/3
L_1_ (high concn)	one	space filling	μ_1_ ≈ μ_2_ ≈ μ_3_ ≈ 1/3
I_1_	many	small	μ_1_ ≈ μ_2_ ≈ μ_3_ ≈ 1/3
H_1_	some	rod-like	μ_1_ ≈ 0, μ_2_ ≈ μ_3_ ≈ 1/2
V_1_/transitional	one	space filling	varied
L_α_	one	slab-like	μ_1_ ≈ μ_2_ ≈ 0, μ_3_ ≈ 1

Calculation of the eigenvalues from the isosurface normals was facilitated with the DL_MESO analysis code^a^ named ISOSURFACES.F90 distributed with the main code. Grid spacing of 0.25 *r*_*c*_ and Gaussian smearing length σ = 0.4 were used to determine isosurfaces.

Determination of system wide equilibration for L_1_ and isotropic phases such as I_1_ and V_1_ does not benefit from monitoring the orientational order parameter as the associated eigenvalues are broadly equal for both these phases (and for randomly orientated “free” surfactant molecules for that matter). For small micelles in the L_1_ region, mean aggregation numbers, *N*_agg_, can be used to monitor equilibration. This typically results from monomer-micelle exchanges in our simulations and tends to occur within ≈1.5 × 10^5^ DPD time units. On this time scale micelle fission and fusion events are infrequent, which could impact the equilibrium micelle sizes (see Appendix B). For identification of the V_1_ and I_1_ phases, visual inspection of the simulated system is required.

### 4.2 Calculating CMC and *N*_agg_

To compare with our earlier work, we also calculated the CMCs and weight-average mean aggregation number (*N*_agg_) for selected surfactants. For this, simulation boxes were constructed such that for each sampled surfactant type, the simulation box contained a total number of surfactant molecules corresponding to four micelles of size indicated by the experimental *N*_agg_ values reported by Swope et al.^21^ In their work Swope and co-workers report *N*_agg_ for various C_*n*_E_*m*_ surfactants at various multiples of the measured CMC. To set the simulation box size as above, we use values of *N*_agg_ for C_8_E_4_, C_10_E_6_, and C_12_E_8_ molecules equal to 153 (0.744 wt %, 3 × CMC), 90 (0.763 wt %, 20 × CMC), and 81.3 (0.295 wt %, 50 × CMC), from the Swope data set, respectively.

As in our earlier work,^1^ we use our standard clustering criterion to identify surfactant aggregates, which we classify as monomers and submicellar aggregates (aggregation number *N* ≤ *N*_cut_) and micelles (*N* > *N*_cut_), using the pragmatic choice *N*_cut_ = 6. The CMC then corresponds to the concentration of “free” surfactant (i.e., monomers and submicellar aggregates), and *N*_agg_ = ∑*N*^2^*p*(*N*)/∑*Np*(*N*), where the sums are restricted to aggregates for which *N* > *N*_cut_, and *p*(*N*) is the aggregation number distribution found by counting aggregates of size *N*. This approach has been widely used in the literature.^1,14,28,38^

These simulations were run for a total of (at least) 4 × 10^5^ DPD time units (40 million steps). The long duration is required to equilibrate *N*_agg_ to a satisfactory level and to ensure appropriate sampling. This is particularly important for the longer tailed molecules studied, which have very long equilibration times in terms of *N*_agg_. CMCs, however, equilibrate relatively rapidly, with the number density of free surfactant taking <3 × 10^4^ DPD time units to reach a steady state. Each system was then monitored to ensure block averages of the free surfactant concentration and *N*_agg_ no longer drift, and the reported CMC value is the mean free surfactant concentration in this steady state region. Appendix B describes the micelle equilibration kinetics in more detail.

### 4.3 Calculating Water–Octanol Partition Coefficients

Values of log *P* were derived using the protocol outlined in Anderson et al.^1^ Briefly, this is a brute-force method in which molecular concentrations are monitored in the emergent octanol-rich and water-rich phases in an elongated single simulation box containing both, starting from a random mixture of water and octanol and a small amount of the molecule for which one wants the log *P* value.

## 5 Results and Discussion

We begin by comparing the model from Anderson et al.^1^ to the revised model developed in this study. In the comparison we explore log *P*, CMC, *N*_agg_, and phase behavior of a small number of nonionic surfactants. We follow this by evaluating the ability of the revised model to reproduce the experimental phase behavior of the nine C_*n*_E_*m*_ surfactants in the C_8_, C_10_, and C_12_ families: specifically C_8_E_4_, C_8_E_5_, C_8_E_6_, C_10_E_5_, C_10_E_6_, C_10_E_8_, C_12_E_6_, C_12_E_8_, and C_12_E_12_.

### 5.1 Original versus Revised Model

The modifications made to the structure of the surfactants by tighter control of bond angles and corresponding angle constants (denoted by θ_0_ and *k*_*a*_), in addition to minor alterations in *A*_*ij*_, were found to have little effect on log *P* for the ether-group-containing small molecules sampled in Anderson et al.^1^Table 4 compares the values obtained using the original and revised models to reported experimental data.

**Table 4:** Calculated Log *P* Values (25 °C) Using the Original Model of Anderson et al.^1^ and the Present Revised Model Compared to Experiment^1^

solute	orig^a^	rev^a^	expt
octanol	3.2(1)	3.2(1)	3.1
water	1.1(1)	1.1(1)	1.3
diglyme	–0.6(1)	–0.4(1)	–0.4
2-hexyloxyethanol	1.7(1)	1.8(1)	1.9
diethyl ether	1.9(1)	2.1(2)	0.9

a Number in parentheses is an estimate of the error in the final digit.

In Table 5, we show the performance of the model of Anderson et al. (hereafter termed the *original* model) in reproducing the experimental water–surfactant phase behavior for C_12_E_6_ and C_8_E_4_ at 25 °C.^1,18^ Associated plots of the resulting eigenvalues versus concentration can be found in the Supporting Information. In the case of C_8_E_4_, the original model results in a stable H_1_ phase in the concentration range 40–50 wt % and an L_α_ phase for the concentrations at approximately 70–80 wt %. This contradicts the known experimental behavior of C_8_E_4_ which shows only an isotropic liquid L_1_ phase across the entire concentration range.^18^ For C_12_E_6_, the original model does a reasonable job of capturing the H_1_ behavior indicated in the experimental phase diagram. The H_1_ phase for this surfactant occurred in simulation in the range 40–60 wt %. At 60 wt % the resultant H_1_ phase was disordered (indicated by “dH_1_” in Table 5) with cylinders meandering through the simulation box, albeit aligned with other cylinders. This behavior persisted to very long simulation times (4 × 10^5^ DPD time units). The experimental phase diagram (Figure 1) indicates a bicontinuous cubic, V_1_ phase for C_12_E_6_ between 63 and 68 wt % which is captured by neither the original nor the revised model (see below). At concentrations of 70 wt % and above, experimentally an L_α_ phase is obtained, which persists until approximately 85 wt %. Further increases in surfactant concentration results in the disappearance of the L_α_ phase and the appearance of an re-entrant isotropic phase (L_1_), before a solid (S) precipitates out at higher concentrations. The original model does not capture the re-entrant L_1_ phase, instead yielding L_α_ behavior to higher concentrations. The revised model behaves as experiment.

**Table 5:** Experimental Phase Behavior^18^ (25 °C) Compared to Results Using the Original Model of Anderson et al.^1^ and the Present Revised Model^a^

	C_8_E_4_			C_12_E_6_		
concn (wt %)	expt	orig	rev	expt	orig	rev
30	L_1_	L_1_	L_1_	L_1_	L_1_	L_1_
40	L_1_	H_1_	L_1_	H_1_	H_1_	L_1_
50	L_1_	H_1_	L_1_	H_1_	H_1_	H_1_
60	L_1_	T	L_1_	H_1_	dH_1_/T	H_1_
65				V_1_	L_α_/T	T
70	L_1_	L_α_	L_1_	L_α_	L_α_	L_α_
80	L_1_	L_α_	L_1_	L_α_	L_α_	L_α_
90	L_1_	L_1_	L_1_	L_1_	L_α_	L_1_

a T represents transitional behavior between two phases.

Overall, the original model demonstrates a reasonable ability to capture the various types of phases formed by C_12_E_6_ but significantly lacks the ability to reproduce correct phase behavior for C_8_E_4_. For the interested reader, in the Supporting Information we present the simulated phase behavior resulting from both the Johnston et al.^22^ and Lavagnini et al.^23^ DPD models for C_8_E_4_ and C_12_E_6_ surfactants.

Further to this, we compared the properties of the micelles at low concentrations in the two models. Table 6 lists the calculated *N*_agg_ and CMC values for C_8_E_4_, C_10_E_6_, and C_12_E_8_ for both models. These values are compared to the experimental values of *N*_agg_ and the modal aggregation number (*N*_mod_) from Swope et al.^21^

**Table 6:** CMCs (wt %), and the Mean (*N*_agg_) and Modal (*N*_mod_) Aggregation Numbers for Three Surfactants of the C_*n*_E_*m*_ Family Compared to the Original Model of Anderson et al.,^1^ the Present Revised Model, and Experimental Values from Swope et al.^21^

surfactant	CMC (expt)	CMC^a^ (orig)	CMC^a^ (rev)	*N*_agg_^a^ (expt)	*N*_mod_ (expt)	*N*_agg_^a^ (orig)	*N*_agg_^a^ (rev)
C_8_E_4_	0.25	0.17(2)	0.25(2)	110(2)	∼40	30(3)	29(2)
C_10_E_6_	0.038	0.06(4)	0.04(3)	90(3)	∼25	44(1)	39(1)
C_12_E_8_	0.0059	0.008(5)	0.007(4)	81.3(3)	∼32	24(1)	26(2)

a The number in parentheses is an estimate of the error in the final digit.

While the CMC from both the revised and original models are in good agreement with experiment, the resulting values of *N*_agg_ warrant further discussion. The calculated values of *N*_agg_ for all three surfactants are in broad agreement with each other for the original and revised models, but both fall well short of the values determined through laboratory experiment.^21^ Low *N*_agg_ values have been reported by a number of authors for various surfactants in both DPD and molecular dynamics studies. For example, in almost all simulation studies of alkyl sulfates, *N*_agg_ is underpredicted.^28,39−41^ This behavior has been attributed to insufficient equilibration since it takes much longer for the micelle size distribution to equilibrate than for the free surfactant (CMC) to reach a steady value.^39,40,42^ The work of Fujimoto and others adds credence to this theory.^43−45^ However, the complexities of equilibrating systems of anionic surfactants, where extensive repulsion between individual micelles can occur, are not present here.

In this study, care was taken to avoid problems with poor equilibration by ensuring that the simulation times were long enough that the number of micelles present in the simulation box was well equilibrated prior to data collection. However, the presence of randomly occurring micelle fusion (and potential fission events) can make determining equilibrium difficult. More details of the calculation of the *N*_agg_ values can be found in Appendix B.

If equilibration is not an issue, a number of alternative possible suggestions exist to explain the low *N*_agg_ values observed here. These include potential sampling issues (simulations are often restricted to a few hundred surfactant molecules at the most) and/or failings in the underlying model and force field. Recently, Johnston et al.^22^ constructed a DPD model for nonionic surfactants where both the CMC and *N*_agg_ values were used as fitting targets. They were better able to reproduce experimental *N*_agg_ values but noted that the aggregation number *distribution* did not match experiment. As a consequence, while the *N*_agg_ values are improved, the *N*_mod_ values are off.

We note that the models in this study give *N*_agg_ values that compare well to the reported *N*_mod_ aggregation numbers reported by Swope, however.^21^ We postulate that the problem here could be in matching the tails of the aggregation number distribution: in simulation we simply do not see the large aggregates that are apparently present in experiment. One possibility is that experimental probes are measuring “aggregates of aggregates”, possibly caused by a many-body interaction between the ether-rich coronas of the micelles. The comparison between simulated and experimental *N*_agg_ is clearly an unresolved and interesting topic for further exploration, but we do not consider this any further in this study.

### 5.2 Revised Model Phase Behavior

All results reported in this section were obtained using the revised model as described above. For the most part we present results in terms of the equilibrated eigenvalues, μ_*i*_, as a function of concentration. These are backed up by visual inspection of the self-assembled mesoscopic structures.

Three C_8_ surfactants were explored with CH_2_OCH_2_ lengths *m* = 4, 5, and 6 which experimentally show only isotropic (L_1_) behavior at 25 °C, although below 15 °C both C_8_E_5_ and C_8_E_6_ display H_1_ behavior at around 60 wt %.^17,18,20^Figure 3 shows the eigenvalues. All three model molecules display the correct isotropic behavior across the concentration range sampled.

The C_10_ surfactants explored in this study display richer phase behavior than the C_8_ surfactants. The resulting eigenvalue versus concentration plots for these surfactants are shown in Figure 4. All show the onset of an H_1_ phase in the region between 40 and 45 wt %. For C_10_E_5_ an L_α_ phase is observed starting at 65–70 wt % and persisting up to 75–80 wt %, above which an isotropic L_1_ phase results. These observations are in good agreement with the available experimental phase diagrams for these surfactants.^46^ For C_10_E_5_ the model overstabilizes the H_1_ phase compared to experiment where this phase melts at about 23 °C. The simulated L_α_ phase of C_10_E_5_ occurs at approximately 5 wt % smaller concentration than experiment but is stable over a similar concentration range of 10 wt %. For C_10_E_6_ the experimental phase diagram shows H_1_ appearing at approximately 45 wt % and persisting until 60–65 wt %, where it switches to an L_1_ phase. This is well captured by the model. Likewise, for C_10_E_8_ the H_1_ phase is well reproduced.

**Figure 6:**
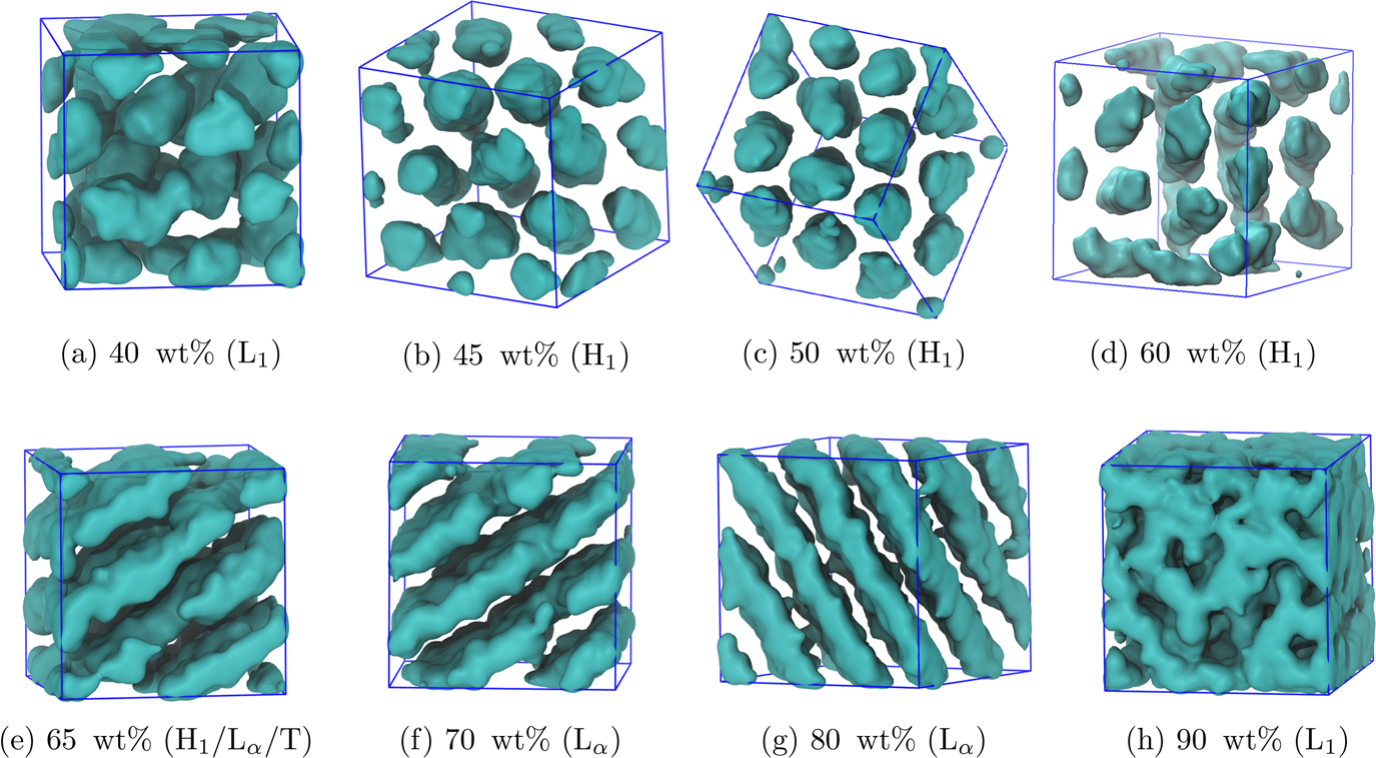
Simulated phase behavior for C_12_E_6_ across the range of concentrations. Only the regions occupied by the alkyl chains have been rendered here. T represents a transitional phase.

Turning now to the C_12_ surfactants, Figure 5 shows the eigenvalues for C_12_E_6_, C_12_E_8_, and C_12_E_12_. For C_12_E_6_ the model does an excellent job at reproducing the experimentally observed phases throughout the sampled concentration range. The H_1_ phase emerges between 40 and 45 wt % which is marginally higher than the corresponding experimental transition at approximately 38 wt %. This phase remains stable to approximately 60 wt % according to the model, at which point branching between rods of the H_1_ phase can be observed. Between 60 and 65 wt %, intriguing behavior is observed in a region close to the experimentally observed V_1_ phase. This region is discussed in detail below. Between 70 and 85 wt % an L_α_ phase is observed which transitions to an L_1_ phase with further increase in concentration. Figure 6 shows the structures of the simulated surfactant phases for C_12_E_6_.

For C_12_E_8_ and C_12_E_12_ the H_1_ phase occurs at higher concentrations than for C_12_E_6_ which is broadly in agreement with the experimental phase diagrams of these surfactants. C_12_E_12_ shows a large I_1_ region in the experimental phase diagram^19^ that spans approximately 30–55 wt %. This behavior is also captured by the model that demonstrates an I_1_ phase between 25 and 55 wt % (determined by visual inspection). The experimental phase diagram of C_12_E_8_ also presents an I_1_ phase between 30 and 45 wt % although only below 15 °C. An L_α_ phase is reproduced for C_12_E_8_ but over a narrower concentration range than for C_12_E_6_ as expected from experimental studies. However, the simulated L_α_ region is larger than that observed in experiment. No evidence of a V_1_ phase was found from the simulations performed for C_12_E_8_. Rather the H_1_ phase transforms to a perforated lamellar phase as the concentration increases.

**Figure 7:**
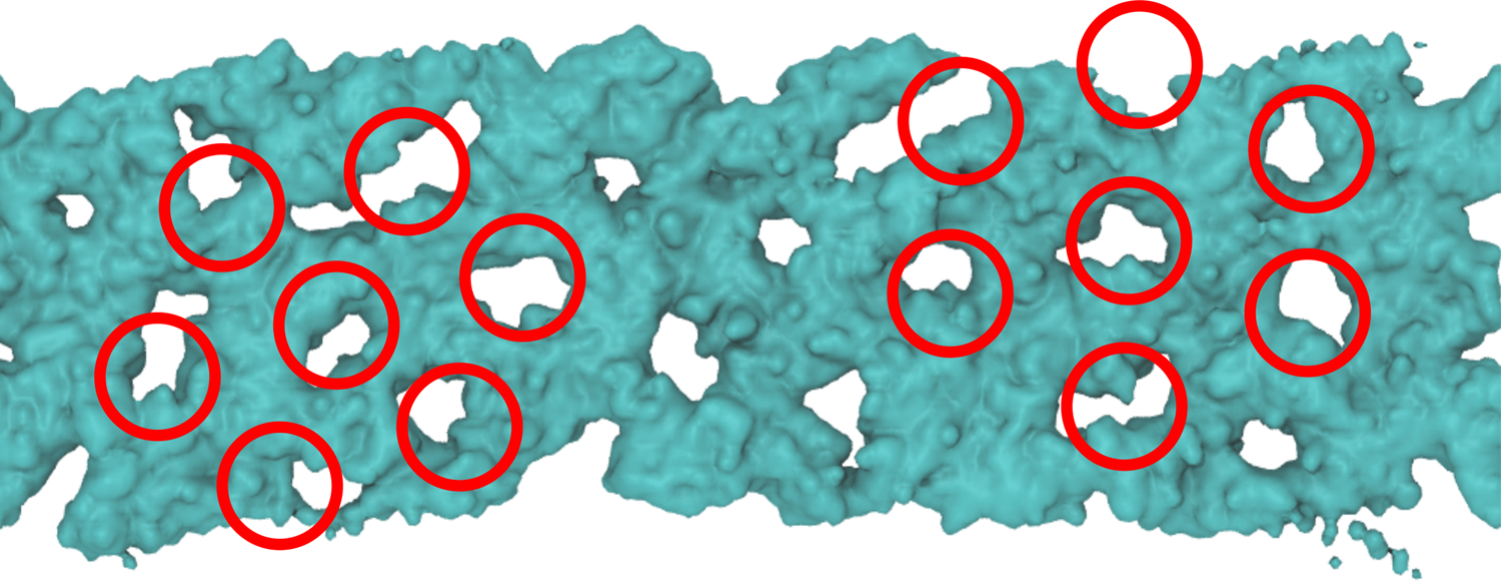
Representative simulation snapshot of hexagonally perforated lamellar structures formed by C_12_E_6_ at 65 wt %. The red circles are a guide to the eye, and again only the regions occupied by the alkyl chains have been rendered.

Returning to the point mentioned above, for C_12_E_6_ there is significant variability in the eigenvalues between 60 and 65 wt %. Simulations were repeated twice in this region, and the eigenvalues for both runs are plotted in Figure 5. Visualization of simulation snapshots in the concentration range 60–64 wt % show semibridged H_1_ structures. At 65 wt % the simulation shows a hexagonally perforated lamellar (HPL) structure (see Figure 7). There have been a handful of studies in the literature describing the emergence of V_1_ and gyroid type structures from HPL structures and noting that the HPL structure is metastable with respect to the formation of gyroid phases.^47,48^ We extended our study to include large simulation boxes (60^3^) and long simulation times (6 × 10^5^ DPD time units) to attempt to resolve the behavior in this region without success.

**Figure 8:**
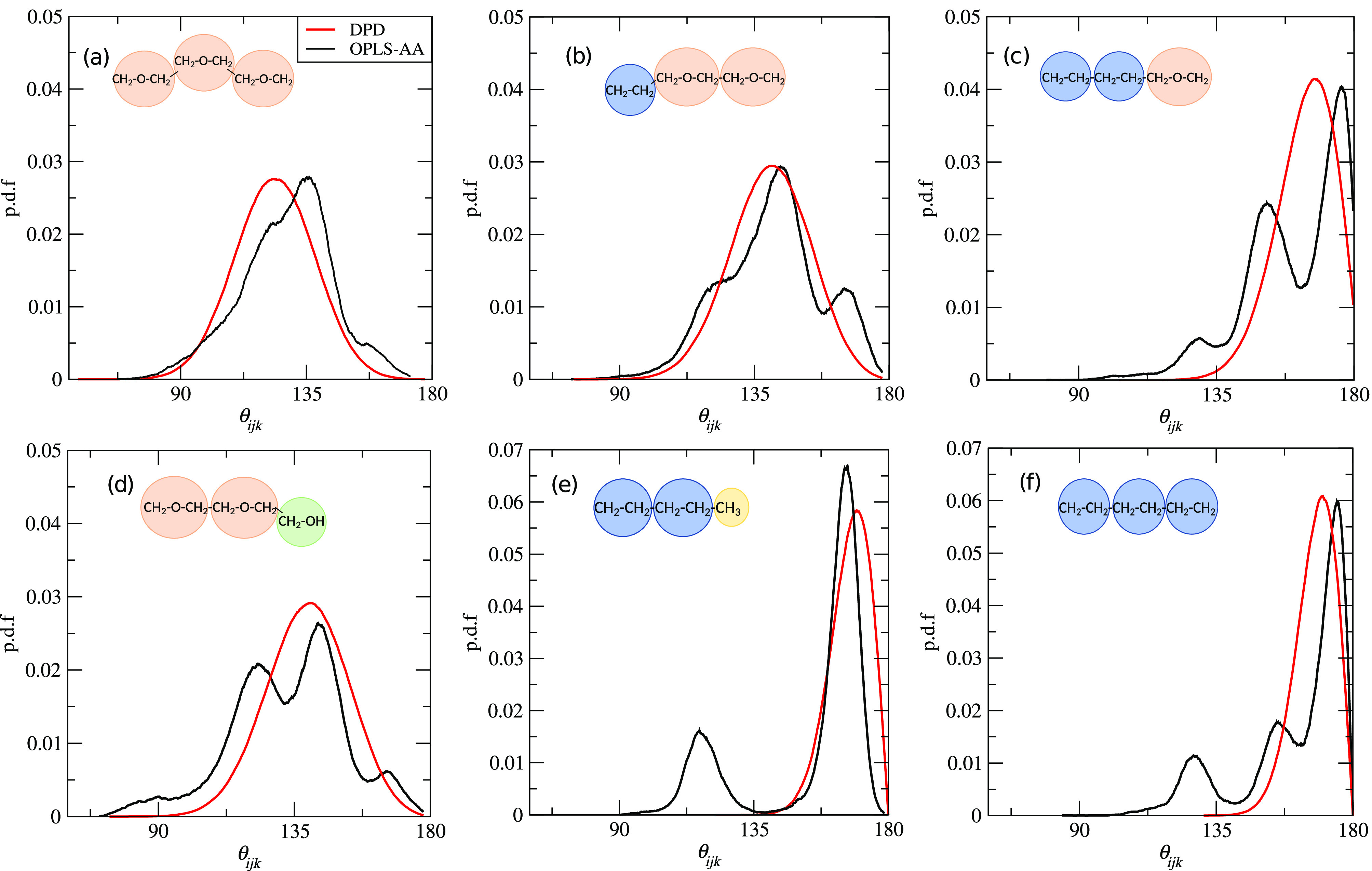
Bond angle distributions from all-atom MD simulations (black lines) and the adopted DPD model (red lines) for the indicated groups.

**Figure 9:**
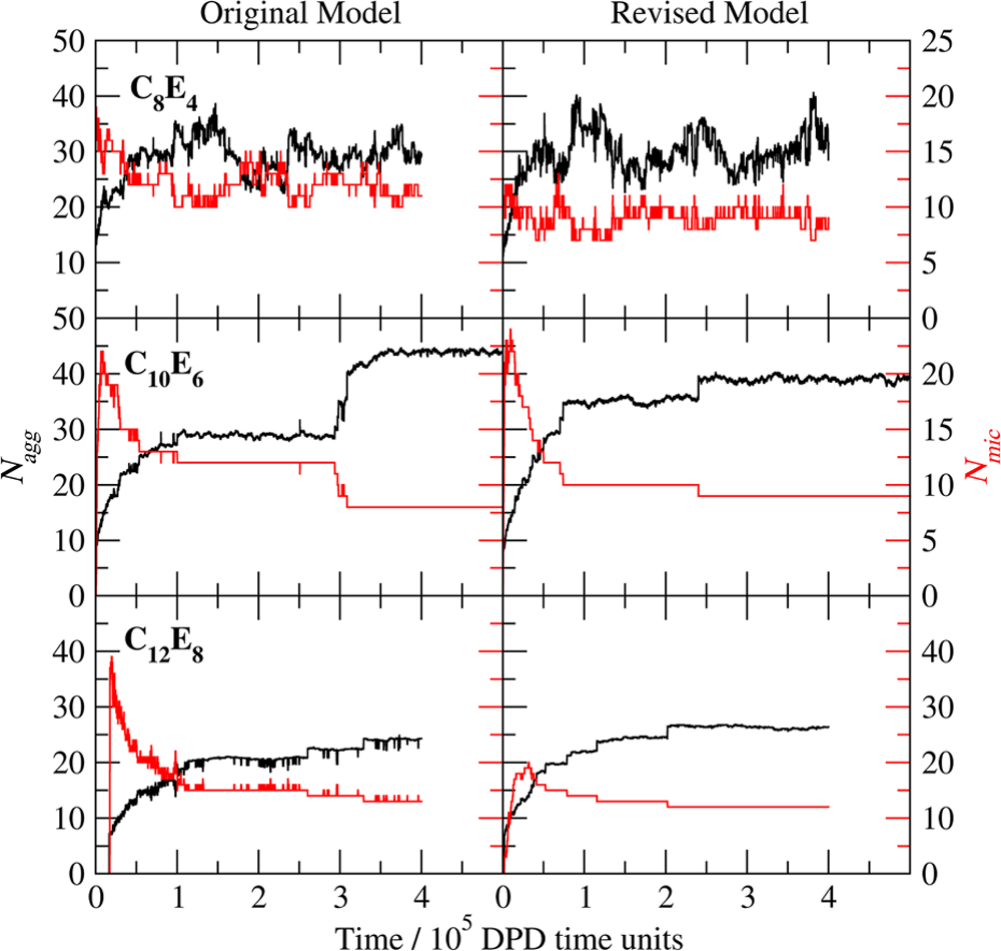
Mean aggregation number, *N*_agg_ (black), and number of micelles, *N*_mic_ (red), for C_8_E_4_ as a function of time (DPD time units) for (a) the original model of Anderson et al.^1^ and (b) the revised model in the present work.

We recognize that using a simulation box with a prespecified size and shape (e.g., cubic) may energetically penalize the formation of phases where the microstructure has a unit cell with dimensions incommensurate with the box. As such, we further explored the region corresponding to the V_1_ phase of C_12_E_6_, trying simulation boxes of different shapes and sizes. Specifically we have sampled the phase behavior of C_12_E_6_ at 65 wt % in boxes with dimensions of 20 × 20 × 67.5 and 40 × 40 × 16.875. Both of these are of the same volume as the original 30^3^ boxes. The phase behavior in these boxes presents as a combination of bridged H_1_ transitioning to perforated L_α_, and vice versa. As we have not been able to fully resolve this region, we therefore refrain from defining it as V_1_ and simply state that for C_12_E_6_ in the range 60–65 wt % there is a transition region between H_1_ and L_α_. Further to the above discussion on box size and shape, we note that the L_1_, I_1_, and L_α_ phases remain stable in simulation boxes that have no dimensions below 25 (for any shape). Below this value, defects can be observed.

When visualizing the simulation snapshots in the adopted 30^3^ simulation boxes for C_10_E_6_, C_10_E_8_, C_12_E_8_, and C_12_E_12_ surfactants, we observe I_1_ type behaviors in the dense micelle regions (i.e., at concentrations just prior to the onset of the H_1_ region). While this behavior is experimentally expected for C_12_E_12_, which has a large I_1_ phase in the 30–50 wt % window, and is present in C_12_E_8_ albeit at below 25 °C, it is more problematic for C_10_E_6_ and C_10_E_8_ because experimentally I_1_ micellar cubic phases are found in neither of these.

This spurious behavior appears to be a consequence of the model not adequately describing the structure of the ethoxylate portion of the surfactants. We have conducted some trial simulations to explore the effects of some key model parameters on these I_1_ regions of the calculated phase diagrams. Table 7 lists some of the combinations of model parameters examined and reports the effect of these changes on the calculated phases of C_10_E_8_ and C_12_E_12_ at 40 wt %. (Beyond these, we found that minor modifications in *k*_a_ (i.e., ± 5) appear to have little effect.) To capture the experimental behavior, C_10_E_8_ should be L_1_ and C_12_E_12_ should be I_1_ at this concentration. We find that for C_10_E_8_ increasing the bond angle between ether beads from θ_0_ = 132° to 140° results in an L_1_ phase. The same effect can be achieved by reducing the ether–ether (nonbonded) repulsion amplitude from *A*_*ij*_ = 25.5 to 22.5 or below. As a final test, we reduced the above bond angle from θ_0_ = 132° to 125° while simultaneously reducing the repulsion amplitude. Here we also recover the desired L_1_ phase. Similar behaviors are observed for C_12_E_12_ upon increasing bond angle and decreasing the repulsion amplitude. For this surfactant the combined reduction in the ether bead bond angle and repulsion amplitude results in the desired I_1_ phase.

**Table 7:** Effect of Changing the Equilibrium Bond Angle θ_0_ and Repulsion Amplitude *A*_*ij*_ for Ether Beads on the Calculated Phase Behavior of the Given Surfactant at 40 wt %

surfactant	θ_0_ (deg)	*A*_*ij*_	phase
C_10_E_8_	132	25.5	I_1_ (cubic)
	140	25.5	L_1_
	132	22.5	L_1_
	132	20.5	L_1_
	125	22.5	L_1_
C_12_E_12_	132	25.5	I_1_ (cubic)
	140	25.5	I_1_ (cubic)
	145	25.5	L_1_
	132	22.5	L_1_
	132	20.5	L_1_
	125	22.5	I_1_ (cubic)

As the behavior of the ethoxylate chain is potentially dependent on a number of coupled elements, we do not attempt to “fix” the model presented here further, e.g., by switching to a different underlying molecular dynamics force field to guide the choice of equilibrium angle, as it is not immediately obvious what combination of changes are most appropriate when factoring in the remainder of the molecule and considering the various properties the model needs to recreate. On the basis of the learnings from the work here and our work on alkane waxes,^2^ it is our opinion that rather than making further *ad hoc* adjustments, a more complete reparametrization is more appropriate, in which we fine-tune the (bonded) structural elements of the model concurrently with the nonbonded interactions.

While the method presented in Section 4.1 for determining the phase behavior, via eigenvalues of the isosurface normals, is not directly applicable to the identification of I_1_ phases, indirect evidence for this phase can be seen in the eigenvalue versus concentration plots for C_10_E_6_, C_10_E_8_, C_12_E_8_, and C_12_E_12_ (Figures 4 and 5). For these surfactants, the eigenvalues remain extremely close in value right up to the formation of the H_1_ phase as micelles remain highly spherical in nature. Contrast this with e.g. C_10_E_5_ or C_12_E_6_ where the eigenvalues begin to diverge as micelles start to elongate with increasing concentration, prior to the formation of the H_1_ phase.

## 6 Conclusions

In this study, we present a DPD model capable of reproducing the lyotropic liquid crystal phase behavior of nine of the alkyl ethoxylate family of nonionic surfactant molecules at *T* = 25 °C. This was achieved by refining the bond angle potentials of a previously published model.^1^ The modification ensured a better surfactant molecular structure representation, similar to the approach reported by Bray et al.^2^ In addition, we tuned the nonbonded DPD repulsion amplitude between the ether and alkane beads (i.e., the head–tail interaction) to prevent undesirable phases from emerging in the calculated phase diagram for the C_8_E_4_ surfactant (which is known to only to display L_1_ behavior by experiment) and to ensure the transition from L_α_ to L_1_ in the high concentration regime of the C_12_E_6_ surfactant is correctly reproduced.^18^ The agreement between experimental phase behavior and our simulated results is remarkable when considering the original interaction parameters (from Anderson et al.^1^) were derived from limited solution state properties, i.e., density and log *P*.

Alteration of the DPD representation of surfactant molecular structure and the change to the bead–bead interaction parameter were demonstrated to have a negligible effect on the log *P* values of small molecules sampled in Anderson et al.^1^ and a minor effect on the CMC and *N*_agg_ values calculated for three surfactant molecules (C_8_E_4_, C_10_E_6_, and C_12_E_8_). Note that while the model seems to be reasonably insensitive to changes in bond angle parameters, we would not expect this to be the case for changes to the pairwise bonding parameters themselves. Modifications to bond length are expected to cause sizable shifts in liquid densities and subsequently log *P* and other observables.

The presented model, however, leaves two open questions:

- While the calculated mean aggregation number values (*N*_agg_) are in agreement with the model of Anderson et al.^1^ they are significantly lower that those reported experimentally by Swope et al.^21^ However, it seems common in simulation to underpredict *N*_agg_ for a variety of different simulation approaches (see Section 5.1).
- The presence of an emergent, spurious, I_1_ phase in the dense micelle regions (typically 35–40 wt %) for C_10_E_6_, C_10_E_8_, and, to a lesser extent, C_12_E_8_ is in contradiction to experiment. This we attribute to subtle deficiencies in modeling the ethoxylate chain. Removal of this I_1_ phase is possible through adjustments to the bonded interactions between ether groups increasing the angles dominating the ethoxylate group and/or reducing the ethoxylate group self-interaction parameter.

For the first of these, work is required to understand the nature of the mismatch in *N*_agg_ to determine, where possible, if the underprediction is a consequence of (a) interaction parameters, (b) the adopted molecular structures, (c) simulation time scales, (d) sampling a small number of surfactant molecules, or (e) a crucial piece of missing physical chemistry (for example, weak many-body interactions between the ethoxylate headgroups).

With regards to the spurious I_1_ phases, we have shown that subtle changes in the bond angles and/or the self-interactions for the ether beads have significant control over resultant phase. While attempts to “fix” or “patch” the model can be made, and indeed were made in this article to better reproduce the phase behavior by modifying the alkyl–ether bead interactions, we prefer to avoid going further in this *ad hoc* direction, favoring instead a more complete reparametrization of the model where the molecular geometry is defined prior to the fitting of the DPD conservative interaction parameters. For this, an attractive approach we are currently investigating is to harness machine learning to accelerate what is traditionally a time-consuming and labor-intensive process.^49^

## Appendix A. Bond Angle Parameters *k*_*a*_ and θ_0_

Specifics of the procedure used to determine θ_*ijk*_ and *k*_*a*_ are presented here. The motivation derives from our recent work exploring freezing transitions (waxing) in alkanes as a function of chain length, where in order to capture the correct behavior significant emphasis was placed on building an accurate representation of the underlying molecular architecture using bond length and angle distributions obtained from molecular dynamics simulation.^2^ Following the same approach for this study, MD simulations of pure systems of alkane, PEO, and C_*n*_E_*m*_ molecules were performed using the optimized potentials for liquid simulation all-atom (OPLS-AA) force field.^50^ The choice of OPLS-AA was motivated by our previous work on alkanes. A study by Fischer et al.^51,52^ compared five force fields to *ab initio* data^53^ on 1,2-dimethoxyethane from which we infer the standard OPLS-AA force field should also be appropriate for the ethylene oxide portion of the surfactant molecules. In these simulations a time step of 2 fs was employed, and the bonds were constrained using the LINCS algorithm.^54^ The van der Waals and Coulomb cutoff was set to 1.1 nm. Long-range electrostatics were calculated using the particle-mesh Ewald summation with a Fourier spacing of 0.12 nm and a fourth-order interpolation. An isothermal–isobaric (NPT) ensemble was used for production run after equilibration simulations. The temperature was maintained at 25 °C with a Nosé–Hoover thermostat and the pressure at 1 bar using a Parrinello–Rahman barostat with a coupling time of 0.1 ps.

From these simulations, bond angle distributions were obtained by calculating the block time average probability density function of angles between the center of masses of the specified groups of atoms. These were then used to match the corresponding bond angle distributions in the coarse-grained DPD model (Figure 8). The values for the angles used in this study are presented in Table 2.

Because of the reduced number of degrees of freedom in the DPD model compared to atomistic model, some compromises have to be made in making these matches. For example, the DPD angle distributions adopted in Figure 8a,b,d reasonably cover the distributions resulting from MD simulation. However, pragmatic decisions have to be made where MD distributions have multiple, distinct peaks in the angle distribution, e.g., Figure 8c,e,f. In these cases one can fit a broad distribution in an attempt to cover all peaks in the distribution or focus on the angles with the highest probability. In the present work we focus mainly on the most probable angles while being mindful to capture as much of the distribution as practically possible with our simple angular potential. The CH_2_OCH_2_–CH_2_OCH_2_–CH_2_OCH_2_ angle used in this work, i.e., 132°, is in good agreement with previously reported values adopted in coarse-grained molecular dynamics models where similar compromises have to be made.^4,31,55^ Note that we do not explicitly attempt to “fit” the DPD distribution to the MD results; rather, we use the latter as a guide while retaining a degree of pragmatism and simplicity in our model.

## Appendix B. Micelle Equilibration Kinetics

While not the main focus of the current article, micelle formation and equilibration kinetics in our coarse-grained surfactant models warrants further discussion, particular with respect to the determination of the mean aggregation number, *N*_agg_. Figure 9 shows how this quantity along with the number of micelles, *N*_mic_, evolves from our choice of a random start (i.e., a “high-temperature quench”), as a function of simulation time, for C_8_E_4_, C_10_E_6_, and C_12_E_8_.

For the C_8_E_4_ models, initial equilibration occurs in approximately 0.5 × 10^5^ DPD time units. Following this initial increase, both *N*_agg_ and *N*_mic_ fluctuate largely in synchrony so that as one increases the other decreases, and vice versa, corresponding to the fact that the number of “free” surfactant molecules has stabilized. For the original (revised) model, an average of 12 (10) micelles are formed. As both simulations start with the same number of surfactant molecules, and the *N*_agg_ values are approximately the same (Table 6), the original model has fewer free monomers and consequently a lower CMC (Table 6).

For C_10_E_6_ the initial equilibration period is longer, taking approximately 10^5^ DPD time units. For both the original and revised models, this initial increase is then followed by a plateau region lasting approximately 1.5 × 10^5^ DPD time units, with the original having a lower aggregation value at this point. Following this, there is a jump in *N*_agg_ for both models; however, the original model has a much larger increase corresponding to a significant reduction in *N*_mic_, likely caused by a micelle fusion event. Here we chose to run simulations for an additional 10^5^ DPD time units to see if the resulting aggregation number would change again. The final section of simulation is again governed by a plateau region where now the original model yields the highest value.

Finally, for C_12_E_8_ we observe an initial equilibration period of 1.5 × 10^5^ DPD time units, then a plateau, and then a jump in *N*_agg_ with a reduction in *N*_mic_. The plot for the original model and the C_12_E_8_ surfactant is much noisier than for the revised model. The cause of this is at present unknown.

These behaviors are in accord with theoretical expectations,^42^ which suggest that the free monomer concentration should relax much more rapidly than the number density of micelles. Indeed, if the kinetics is dominated by monomer–micelle exchange events as in the Aniansson–Wall model,^42^ then the micelle number density is a quasi-conserved quantity because gaining or losing monomers does not change the number of micelles. The only processes that can change the latter are the rare stepwise complete assembly or disassembly of a micelle from individual monomers, or micelle fission and fusion events not included in the theory. Experimentally, this results in two distinct time scales in the relaxation kinetics of a surfactant solution: *t*_1_ [ ≳ *O*(1 μs)] ≪ *t*_2_ [ ≳ *O*(1 ms)] (typical values) corresponding to respectively monomer–micelle exchange events and the relaxation of the quasi-conserved micelle number density.^42,56^ This is similarly so in our simulations as indicated in the plots in Figure 9. While we anticipate having perhaps many hundreds of monomer–micelle exchange events, the rarity of processes that change the number of micelles is clearly demonstrated by the long quasi-steady-state plateaus in *N*_agg_ and *N*_mic_, punctuated by the occasional rare event which causes these quantities to jump.

In reporting *N*_agg_, we adopt a pragmatic approach and use values from the final plateau region for each simulation. This means that the period for which these quantities are calculated over differs for each molecule and model. For the C_8_E_4_ surfactants, the final time window for calculating *N*_agg_ is (2–5) × 10^5^ DPD time units. For C_10_E_6_ original, we calculate *N*_agg_ after 3.75 × 10^5^ DPD time units and for the revised model after 2.5 × 10^5^ DPD time units. For C_12_E_8_ original we use the final 0.75 × 10^5^ DPD time units, whereas the corresponding revised model is determined over the final 1.5 × 10^5^ DPD time units.

Without undertaking “steered” computational simulations in which the rare processes are artificially promoted, one way to assess the fluctuations in these quantities would be to undertake replicate simulation runs, but even this assumes that there is no lingering effect of the choice of random initial conditions. However, the study of these systems on such large time scales is computationally intensive. For example, to run out to 5 × 10^5^ DPD time units (50 million steps) of the C_12_E_8_ systems required approximately 10 days of compute time on 256 cores of our Atos BullSequana X1000 system.

Given the apparent mismatch between experimental and simulated *N*_agg_ values presented in this work and the different possibilities leading to this as discussed in the main text, a detailed study of micelle formation and equilibration kinetics for these coarse-grained surfactant models is clearly warranted but beyond the scope of the present work.

The above results are reported in DPD time units, and the curious reader may wish to know if there is a conversion to “real” time. For this, we calibrated the time scale by measuring the self-diffusion coefficients of a few surfactants (unpublished) obtaining an estimate of 50 ps for the DPD time unit. This is comparable to a DPD model developed by Fraaije et al.,^27^ with a similar level of coarse-graining and molecular fragmentation strategy, for which they report a DPD time unit of 64 ± 13 ps using a correlation to experimental diffusion coefficients of small molecules.^57^ In the present model therefore 10^5^ DPD time units corresponds to an elapsed time of 5 μs, and the above micellar relaxation times are of this same order of magnitude. For example, for C_8_E_4_ (Figure 9), if the time to reach equilibrium is (2–3) × *t*_1_, one might estimate *t*_1_ ≈ 0.2 × 10^5^ DPD time units ≈1 μs. This is exactly on top of the experimental value (*t*_1_ = 1.0 μs) reported by Kato et al.^56^ for C_8_E_4_ at about 2 × CMC using an ultrasound method.^58^ While suggestive and encouraging, we have not attempted to quantify this further because a detailed study of surfactant micellization kinetics is rightly the subject of separate study (work in progress).

## Supporting Information Available

Volumes of the DPD beads used in this study; details on determining phase behavior from visualization of simulated morphology; a brief study corresponding to simulating solid crystalline behavior of certain surfactants; an assessment of the phase behavior for C_12_E_6_ and C_8_E_4_ determined using DPD interaction parameters from refs (22) and (23) with the methods outlined in the main article; plots of the eigenvalues of the orientational order parameter for C_12_E_6_ and C_8_E_4_ resulting from the model of Anderson et al.^1^ in support of Table 5 and the associated discussion (PDF)

## Footnotes

a UMMAP is also capable of determining eigenvalues of the isosurface normals and the values are equivalent to DL_MESO.
