# Supplementary material for: Phase Behavior of Alkyl Ethoxylate Surfactants in a Dissipative Particle Dynamics Model
Source: J Phys Chem B. 2023 Feb 14;127(7):1674–87. doi: 10.1021/acs.jpcb.2c08834 (PMC9969514; doi:10.1021/acs.jpcb.2c08834)
Supplement: Supplementary file 1 — jp2c08834_si_001.pdf [file jp2c08834_si_001.pdf]

Phase Behaviour of Alkyl Ethoxylate Surfactants in a Dissipative Particle Dynamics Model

Richard L. Anderson,* David S. D. Gunn, Tseden Taddese, Ennio Lavagnini, Patrick B. Warren, and David J. Bray

The Hartree Centre, STFC Daresbury Laboratory, Warrington, WA4 4AD, United Kingdom

E-mail: richard.anderson@stfc.ac.uk

1 Bead Volumes Corresponding to the DPD Model

Table S1 presents the bead volumes for the chemical species studied in the main article.

Table S1: Bead Radius (DPD units) and Molar Volumes ($cm^3\,mol^{-1}$) Corresponding to the DPD Beads Calculated Using the Rules Presented by Durchschlag and Zipper[1] for Species Studied in the Main Article.

bead	R_{ij}	molar volume
$2\,H_2O$	1.0000	36.0
CH_3	0.9570	31.6
CH_2CH_2	1.0740	44.6
CH_2OCH_2	1.1160	50.1
CH_2OH	0.9800	33.9

2 Regions Denoted 'S' in the Phase Diagrams

For temperatures below the melting point of the pure surfactant, the Krafft boundary indicates the solubility limit in water for the solid surfactant, and the region below this is denoted by 'S' in the phase diagram. For a state point in such a region, phase coexistence obtains between solid surfactant and the appropriate liquid phase. As the chain lengths increase, the melting points of the alkyl ethoxylate surfactants also increase, and consequently the Krafft boundary moves up in temperature and the 'S' region expands. For example, pure $C_{12}E_{12}$ melts at approximately 40 ℃ (see Fig. 5 in the main text) and the Krafft boundary is at approximately 87 wt% at 25 ℃; to the right of this is the 'S' region. This means that in this system for instance, one should expect to see precipitation or freezing out of the pure surfactant as a molecular crystal in this region. As a prerequisite, this demands of the model that the pure surfactant should freeze at 25 ℃.

Here we report on work undertaken to understand the limitations of the model from the main text in this respect, focussing on $C_{12}E_{12}$. We explore multiple options relating to the modification of our model to begin to explore what factors lead to surfactant freezing.

Analysis is carried out using protocols developed in our previous work for freezing (waxing) of alkanes.[2] These protocols involve monitoring a nematic order parameter and mean squared displacement (MSD) of the molecules over a time interval $\Delta t = 500$ DPD time units, for identifying the crystalline phase (see Bray *et al.*[2] for details). We additionally assessed the extent of segregation which occurred between the alkyl and ethoxylate groups which appears to be a necessity for locking of molecules into the solid. We explore four options for the bonded and non-bonded interactions of $C_{12}E_{12}$:

(Base) The parameter set detailed in the main text as the *revised model.*

(Rigid) Bond stiffness increased to $k_b = 5000\, k_{\mathrm{B}}T$ and corresponding r_0 increased to maintain constant equilibrium length (as prescribed by Bray *et al.*[2]).

(Straight) All equilibrium angles set as $\theta_0 = 180°$ to straighten out the ethoxylate chain.

(Params) A return to the Anderson *et al.*[3] A_{ij} parameter values (k_a are unchanged).

Note in each case our *revised model* provides the base model parameters which are then modified as *per* the additional criteria. From the above options, nine different combinations were tried, with results listed in Table S2. Adding the *Straight* option produces rod like molecular structures akin to the alkane models studied in Bray *et al.*[2]. Whilst not consistent with our model in the main article we wished to explore potential implications resulting from geometrical conditions.

Table S2: Test Models Used for Studying the Freezing of Pure $C_{12}E_{12}$, the Resultant Properties and Assigned State (I – Isotropic Liquid, N – Nematic Liquid Crystal, X – Crystalline Solid).

Test model	Rigid bonds	Linear EO chain	Anderson *et al.*[3]	Segregated	Nematic order	MSD	Phase
Base	N	N	N	no	low	high	I
Rigid	Y	N	N	no	low	high	I
Params	Y	Y	Y	yes	low	moderate	I
Params *	N	Y	Y	yes	low	moderate	I
Rigid + Params	Y	N	Y	yes	low	moderate	I
Straight	N	Y	N	no	high	high	N
Rigid + Straight	Y	Y	N	no	high	high	N
Straight + Params	N	Y	Y	yes	high	low	X
Rigid + Straight + Params	Y	Y	Y	yes	high	low	X

* Bond angles weakened to $k_a = 5\,k_\mathrm{B}T$

(a) (b)

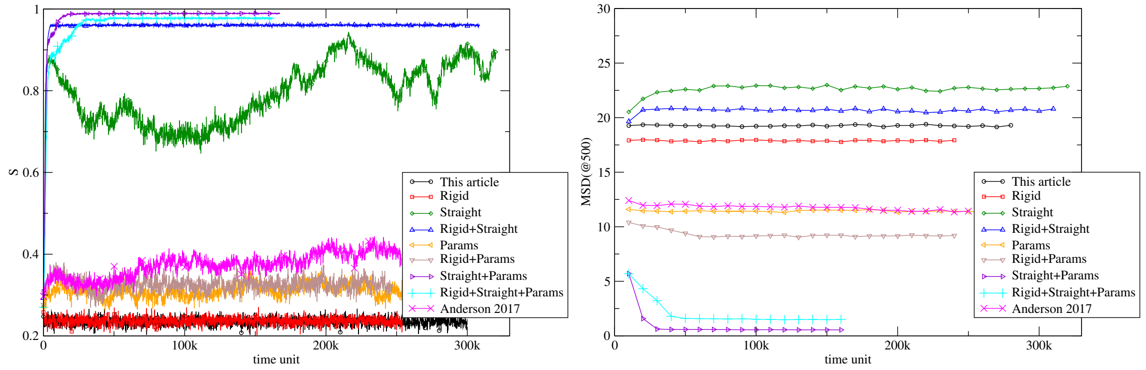

Figure S1: Pure $C_{12}E_{12}$ simulations: time evolution of (a) the nematic order parameter and (b) mean square molecular displacement over an interval Δt = 500 DPD time units (see Bray *et al.*[2] for details), for the model variants in Table S2.

Figures S1 present the system wide order and MSD corresponding to each of the scenarios above. Figures S2 show snapshots resulting from the performed simulations. We ran the simulations up to 300,000 DPD time units using the same conditions as described in the main article. The results show that the choice of parameters and angle have the biggest effect on whether a solid is produced. The *revised model* presented in the main article produced an isotropic liquid with low order, high molecular diffusion and no segregation (Figure S2(a)). We were unable to force freezing, or segregation of alkyl and ethoxylate beads, of the surfactant using this model by altering the bond stiffness (Rigid), geometry (Straight), see Figures S2(a-d). Driven by curiosity we explored the ability of the *original model* to reproduce the freezing behavior. The Anderson *et al.*[3] regains the $C_{12}E_{12}$'s ability to segregate (clusters of red and grey in the figure), Figures S2(e-i)). Straightening the molecules results in high alignment with neighbouring molecules (Figures S2(c-d,g-h)). Both these two requirements seem to be necessary to cause the molecular diffusion to drop and the crystal to form (which occurred within 50,000 DPD time units, see Figures S2(g,h)). Bond rigidity appears to be of secondary importance and leads to more stable conformations with lower molecular diffusion than the more flexible case (Figures S2(b,d,f,h)).

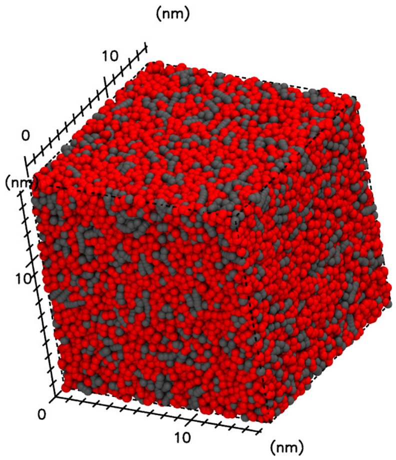

(a) Base ($t = 299,900$)

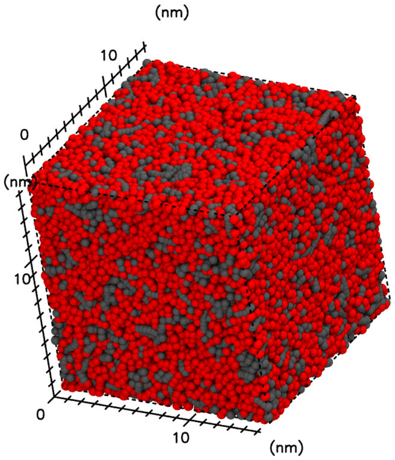

(b) Rigid ($t = 254,000$)

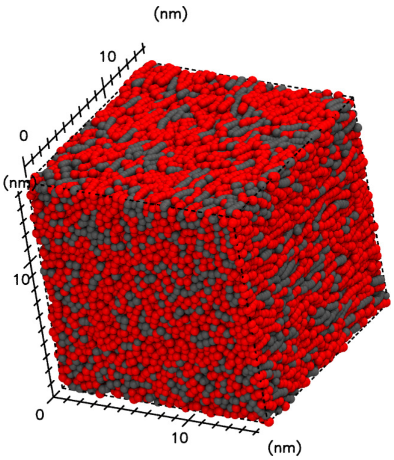

(c) Straight ($t = 314,200$)

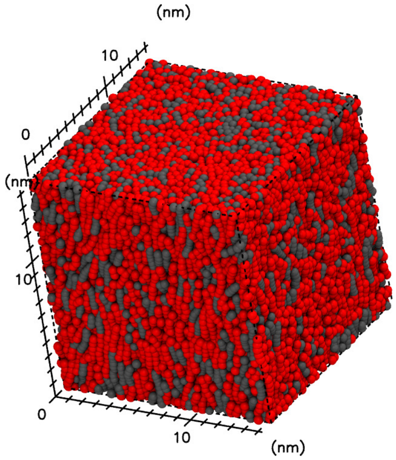

(d) Rigid + Straight ($t = 308,700$)

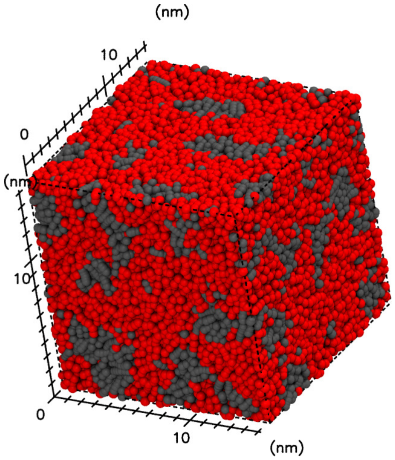

(e) Params ($t = 282,500$)

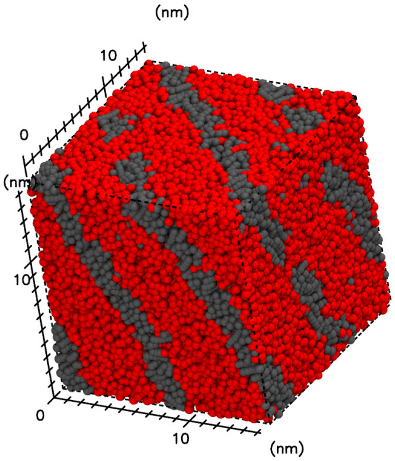

(f) Rigid + Params ($t = 247,100$)

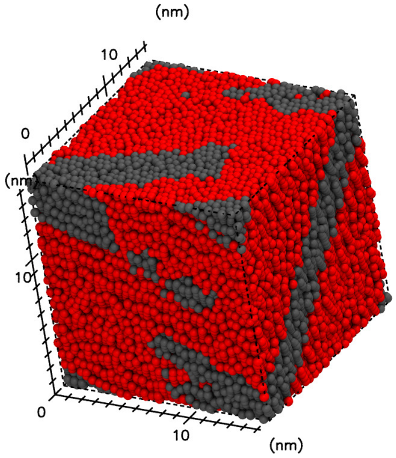

(g) Straight + Params ($t = 167,400$)

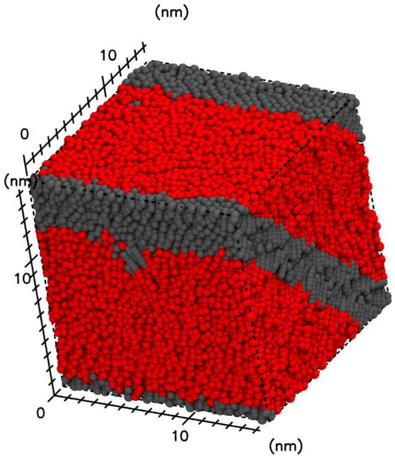

(h) Rigid + Straight + Params ($t = 162,100$)

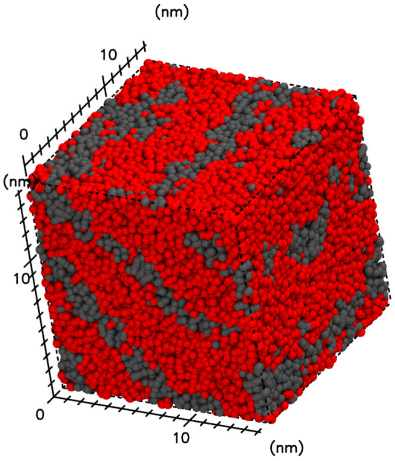

(i) Params, with bond angles as $k_a = 5\,k_{\mathrm{B}}T$ ($t = 339,300$)

Figure S2: Snapshots of the *final* state (time t), for the model variants of $C_{12}E_{12}$ in table S2. alkyl and ethoxylate beads are shown in grey and red respectively.

3 Catalog of Phase Morphologies

In this section we present a catalog of the typical phase morphologies seen in simulation for the different phases encountered in the main article. Identifying phases from static images can often be challenging so we provide rough descriptions and comments where appropriate. We hope the typical images presented here will aid future work.

Image	Phase	Description	Comments
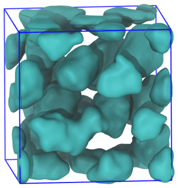	L_1 (low concentration)	Typical presentation is multiple aggregates of low aspect ratio.	L_1 also corresponds to high concentration large disordered aggregates (see below).
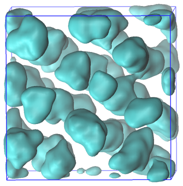	I_1	Many aggregates of the same size aligned along a particular direction.	May not be completely aligned, look for order in the simulation box.
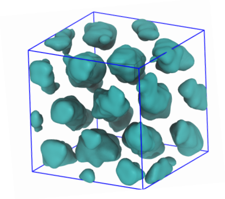	H_1	A series of tubes/rods aligned along a particular direction.	There can often be bridging between rods and some defects in the structure.
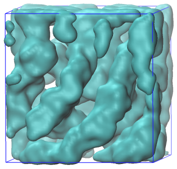	V_1 / T	A space filling phase which presents similarly to the high concentration L_1 phase, sometimes displaying order.	Usually presents with increased segregation in the eigenvalues of the isosurface normals than the L_1 phase.
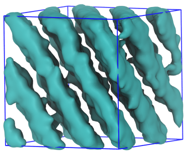	L_α	Easy to identify as regular ordered slabs in the simulation cell.	May present as bridged or perforated lamellar.
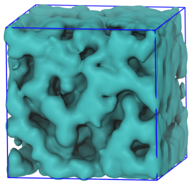	L_1 (high concentration)	Isotropic space filling phase.	Isosurface normal eigenvalues will all be broadly equal.

Figure S3: Typical Simulation Snapshots Corresponding to Different Phases Encountered in the Main Article.

4 Phase Diagrams of C_8E_4 and $C_{12}E_6$ According to the Johnston *et al.*[4] Model.

Here we present the phase diagrams of $C_{12}E_6$ and C_8E_4 as determined by the DPD model for non-ionic surfactants developed by Johnston and co-workers (Figures S4 and S5).[4] In this model, the C_nE_m molecules are governed by only two bead types (CH_2CH_2 & OCH_2CH_2) versus the four adopted by the model presented in the main article. Specifically, the Johnston model does not incorporate the terminal polyethylene oxide (PEO) chain functionality of CH_2OH and their specification of the PEO chain beads are subtly different, preferring to adopt beads of OCH_2CH_2 versus the CH_2OCH_2 we adopt in our work. This difference facilitates our model incorporating the CH_2OH and CH_3 terminal beads as presented in the main article. Parameters defining the Johnston model can be found in their original article.

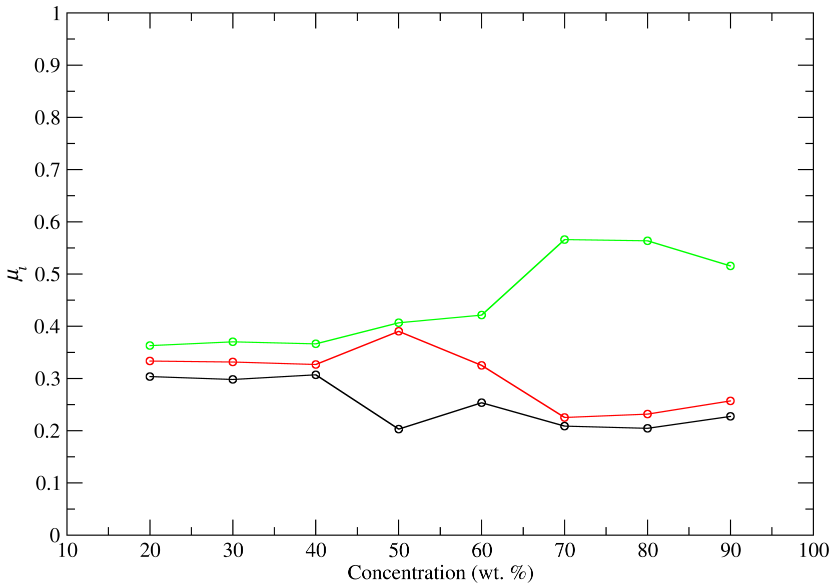

Figure S4: Eigenvalues of the second moment of the isosurface normal distribution, as a function of concentration, for $C_{12}E_6$, determined via simulations using the Johnston *et al.*[4] DPD model. Lines are μ_3 (green - top), μ_2 (red - middle) and μ_1 (black - bottom).

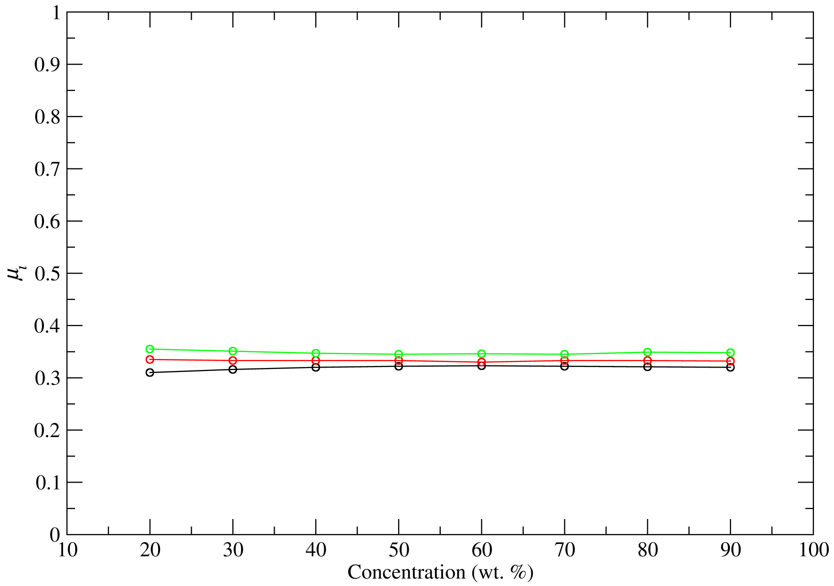

Figure S5: Eigenvalues of the second moment of the isosurface normal distribution, as a function of concentration, for C_8E_4, determined via simulations using the Johnston *et al.*[4] DPD model. Lines are μ_3 (green - top), μ_2 (red - middle) and μ_1 (black - bottom).

Simulations were set up at 10 wt% spacing and, as in the main text, we sample over 20 million steps whilst applying the same phase identification criteria as we have throughout. The Johnston model results in phase behavior well aligned to the experimental phase diagrams of the sampled surfactants. Here the Johnston model yields a single L_1 phase across the concentration ranged scanned for C_8E_4 and manages to reproduce the L_1, H_1, and L_α phases of $C_{12}E_6$. For $C_{12}E_6$ the Johnston model presents a transitional region at 60 wt% that appears as a perforated lamellar phase when visualising the outputs of simulation. The H_1 phase appears to be slightly narrower than the experimental phase behavior although it would be prudent to sample the phase behavior with more simulated points to be certain. The L_α phase extends to a too high concentration where experimentally this phase would transition to L_1 at approx 85 wt%.

5 Phase Diagrams of C_8E_4 and $C_{12}E_6$ According to the Lavagnini *et al.*[5] Model.

Here we present the phase diagrams of $C_{12}E_6$ and C_8E_4 as determined by the DPD model for non-ionic surfactants developed by Lavagnini and co-workers (Figures S6 and S7).[5] In this model, the molecules are governed by the same bead types as in the main article. Parameters defining the Lavagnini model can be found in their original article. Simulations were set up at 10 wt% spacing and, as in the main text, we sample over 20 million steps whilst applying the same phase identification criteria as we have throughout. The Lavagnini model results in phase behavior well aligned to the experimental phase diagrams of the sampled surfactants. Here the Lavagnini model yields a single L_1 phase across the concentration ranged scanned for C_8E_4 and manages to reproduce the L_1, H_1, and L_α phases of $C_{12}E_6$.

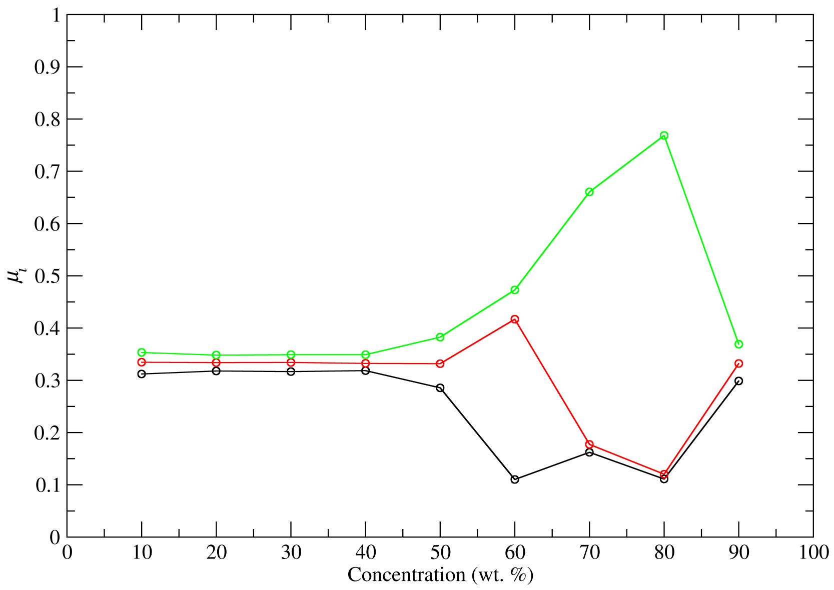

Figure S6: Eigenvalues of the second moment of the isosurface normal distribution, as a function of concentration, for $C_{12}E_6$, determined via simulations using the Lavagnini *et al.*[5] DPD model. Lines are μ_3 (green - top), μ_2 (red - middle) and μ_1 (black - bottom).

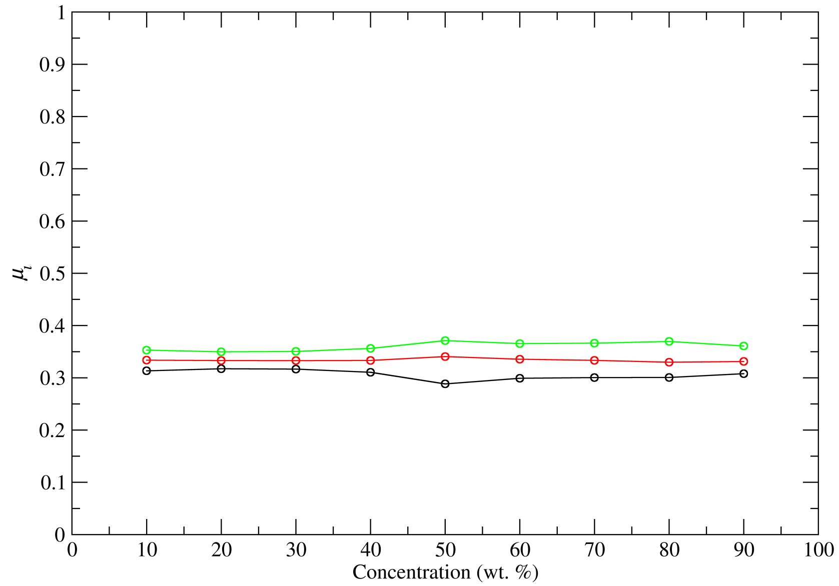

Figure S7: Eigenvalues of the second moment of the isosurface normal distribution, as a function of concentration, for C_8E_4, determined via simulations using the Lavagnini *et al.*[5] DPD model. Lines are μ_3 (green - top), μ_2 (red - middle) and μ_1 (black - bottom).

The onset of the H_1 phase of $C_{12}E_6$ appears to occur at a higher concentration than reported experimentally and is narrower in it's stability in terms of concentration range. The onset of the L_α phase is in line with that expected from experimental studies and transitions to a re-entrant L_1 phase between 80 and 90 wt% as expected. For $C_{12}E_6$ the Lavagnini model may present a transitional region at approx 65 wt%, however, we did not sample at this point in our brief screen of phase behavior of this surfactant.

6 Phase Diagrams of C_8E_4 and $C_{12}E_6$ According to the Original Model of Anderson *et al.*[3]

Here we present the plots of concentration versus the eigenvalues of the orientational order parameter **M** for the $C_{12}E_6$ and C_8E_4 surfactants. These images support the data presented in Table 5 of the main article and the discussion therein.

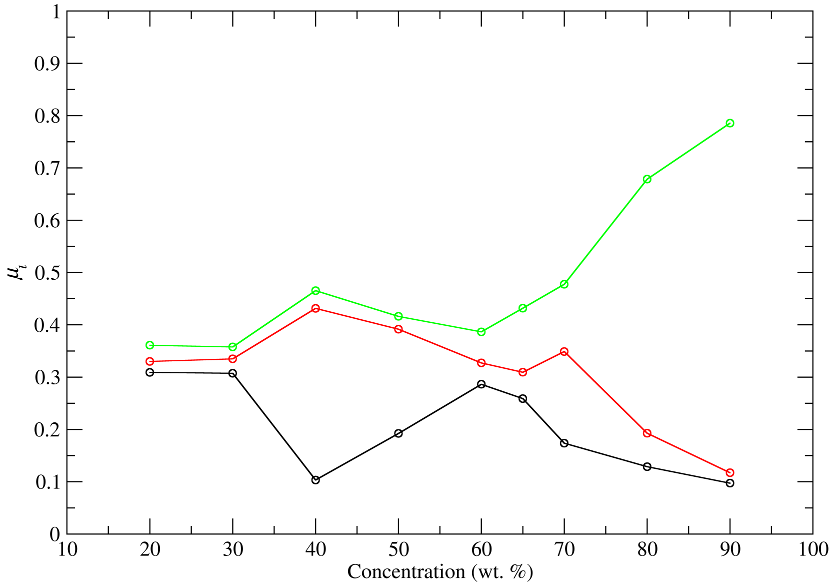

Figure S8: Eigenvalues of the second moment of the isosurface normal distribution, as a function of concentration, for $C_{12}E_6$, determined via simulations using the Anderson *et al.*[3] DPD model. Lines are μ_3 (green - top), μ_2 (red - middle) and μ_1 (black - bottom).

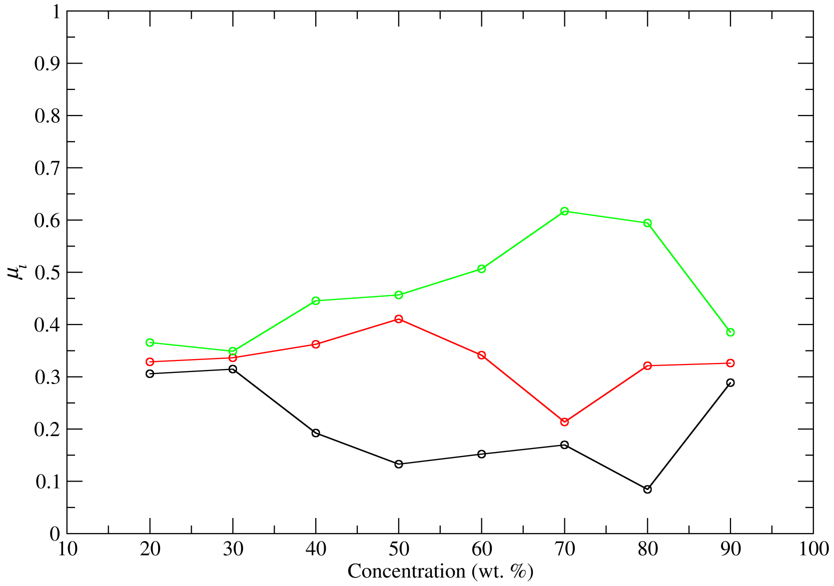

Figure S9: Eigenvalues of the second moment of the isosurface normal distribution, as a function of concentration, for C_8E_4, determined via simulations using the Anderson *et al.*[3] DPD model. Lines are μ_3 (green - top), μ_2 (red - middle) and μ_1 (black - bottom).

References

(1) Durchschlag, H.; Zipper, P. Calculation of the partial volume of organic compounds and polymers. *Prog. Colloid. Polym. Sci.* **1994**, *94*, 20–39.

(2) Bray, D. J.; Anderson, R. L.; Warren, P. B.; Lewtas, K. Wax formation in linear and branched alkanes with dissipative particle dynamics. *J. Chem. Theory Comput.* **2020**, *16*, 7109–7122.

(3) Anderson, R. L.; Bray, D. J.; Ferrante, A. S.; Noro, M. G.; Stott, I. P.; Warren, P. B. Dissipative particle dynamics: Systematic parametrization using water-octanol partition coefficients. *J. Chem. Phys.* **2017**, *147*, 094503.

(4) Johnston, M. A.; Duff, A. I.; Anderson, R. L.; Swope, W. C. Model for the simulation of the C_nE_m nonionic surfactant family derived from recent experimental results. *J. Phys. Chem. B* **2020**, *124*, 9701–9721.

(5) Lavagnini, E.; Cook, J. L.; Warren, P. B.; Williamson, M. J.; Hunter, C. A. A surface site interaction point method for dissipative particle dynamics parametrization: application to alkyl ethoxylate surfactant self-assembly. *J. Phys. Chem. B* **2020**, *124*, 5047–5055.
